# A Novel Role for Wnt/Ca^2+^ Signaling in Actin Cytoskeleton Remodeling and Cell Motility in Prostate Cancer

**DOI:** 10.1371/journal.pone.0010456

**Published:** 2010-05-04

**Authors:** Qin Wang, Andrew J. Symes, Corrina A. Kane, Alex Freeman, Joseph Nariculam, Philippa Munson, Christopher Thrasivoulou, John R. W. Masters, Aamir Ahmed

**Affiliations:** 1 Prostate Cancer Research Centre and Division of Surgery, University College London, London, United Kingdom; 2 Department of Histopathology, University College Hospitals London National Health Service Foundation Trust, London, United Kingdom; 3 University College London Advanced Diagnostics, University College London, London, United Kingdom; 4 The Centre for Cell and Molecular Dynamics, University College London, London, United Kingdom; University of Birmingham, United Kingdom

## Abstract

Wnt signaling is a critical regulatory pathway in development and disease. Very little is known about the mechanisms of Wnt signaling in prostate cancer, a leading cause of death in men. A quantitative analysis of the expression of Wnt5A protein in human tissue arrays, containing 600 prostate tissue cores, showed >50% increase in malignant compared to benign cores (*p*<0.0001). In a matched pair of prostate cancer and normal cell line, expression of Wnt5A protein was also increased. Calcium waves were induced in prostate cells in response to Wnt5A with a 3 fold increase in Flou-4 intensity. The activity of Ca^2+^/calmodulin dependent protein kinase (CaMKII), a transducer of the non-canonical Wnt/Ca^2+^ signaling, increased by 8 fold in cancer cells; no change was observed in β-catenin expression, known to activate the canonical Wnt/β-catenin pathway. Mining of publicly available human prostate cancer oligoarray datasets revealed that the expression of numerous genes (e.g., CCND1, CD44) under the control of β-catenin transcription is down-regulated. Confocal and quantitative electron microscopy showed that specific inhibition of CaMKII in cancer cells causes remodeling of the actin cytoskeleton, irregular wound edges and loose intercellular architecture and a 6 and 8 fold increase in the frequency and length of filopodia, respectively. Conversely, untreated normal prostate cells showed an irregular wound edge and loose intercellular architecture; incubation of normal prostate cells with recombinant Wnt5A protein induced actin remodeling with a regular wound edge and increased wound healing capacity. Live cell imaging showed that a functional consequence of CaMKII inhibition was 80% decrease in wound healing capacity and reduced cell motility in cancer cells. We propose that non-canonical Wnt/Ca^2+^ signaling via CaMKII acts as a novel regulator of structural plasticity and cell motility in prostate cancer.

## Introduction

Wingless/Wnt genes code for a family of secreted glycoproteins that regulate many cellular processes [1], [2]. Aberrant signaling through Wnt signaling pathways is linked to a number of diseases including cancer [3]–[6]. Wnt signaling occurs via the canonical (Wnt/β-catenin, CTNNB1) and non-canonical (Wnt/Ca^2+^) pathways [3], [7]. A less well described Wnt pathway is the Wnt-JNK pathway [8]. Signaling via the Wnt/β-catenin pathway activates transcription of many genes, such as cyclin Ds and membrane metalloproteinases (MMPs), via TCF/LEF factors. Wnt/Ca^2+^ pathway leads to an increase in intracellular calcium [9] and activation of calmodulin dependent protein kinase II (CaMKII) [10] and is known to modulate cell movement and behavior [11], [12] and induces structural changes [11], [13], [14]. The link between increased β-catenin signaling in tumorigenesis and increased transcription of genes involved in cell transformation and proliferation are documented, however, the role non-canonical Wnt/Ca^2+^ signaling in cancer is only slowly being elucidated [15], [16].

Prostate cancer accounts for an estimated 25% of all new cancer cases and is the second leading cause of cancer deaths in males [17]. Neither the expression of Wnt proteins nor the mechanisms of Wnt signaling in prostate cancer are understood [18]. Previous studies concentrated on characterization of Wnt receptors, receptor related proteins and expression of Wnt genes [5], [19]–[21], in prostate cancer cell lines. Direct interaction between androgen receptor (AR) and TCF for β-catenin and other transcription co-factors was also suggested [22]. However, only a small percentage of prostate cancer samples have dysregulated destruction complex or mutated β-catenin [18]. It is likely therefore that other mechanisms, perhaps directly linked to Wnt signaling, may be involved.

We recently showed [23] that WNT5A (one of the 19 members of Wnt family) gene expression is increased (>50 fold) in prostate cancer tissue and cancer cell lines due to hypomethylation. However, important questions regarding Wnt5A expression and the mechanisms of Wnt5A mediated signaling in prostate cancer remain. For example, we do not know (i) Whether the increase in WNT5A gene transcription results in increased Wnt5A protein expression in malignant tumors? (ii) Whether the canonical or non-canonical signaling pathway is activated in prostate cancer? (iii) What is the functional role of Wnt signaling in prostate cancer? To address these questions we tested the following hypotheses: (i) That Wnt5A protein expression is increased in human prostate cancer (ii) That Wnt/β-catenin signaling should result in increased expression of TCF/LEF target genes such as MMPs and TIMP3 in prostate cancer (iii) Activation of Wnt/Ca^2+^ signaling in prostate cancer cells should result in an increase in CaMKII enzyme activity causing alterations in cytoskeleton and cell motility. We show, for the first time, that Wnt5A protein expression is increased in malignant human prostate compared to benign tissue and Wnt/Ca^2+^ signaling mechanism is activated in prostate cancer cells. We also provide novel mechanistic evidence of a critical role for CaMKII as a modulator of actin cytoskeleton and cell motility in prostate cancer.

## Results

### RNA transcript assessment using real time PCR

We measured the mRNA levels for WNT5A (Table S1), previously identified from our oligoarray analysis to be dysregulated in prostate cancer cells [23]. WNT5A mRNA displayed a similar magnitude of change (∼50 fold) in 1542-CP_3_TX cancer cells compared to normal 1542-NPTX cells, whether measured using oligoarray [23] or real time PCR (Table S1). WNT5A transcript is also expressed in other prostate cancer cell lines e.g. PC3 and DU145 [19], [23].

### Wnt5A protein expression in malignant and benign human prostate

**Figure 1:**
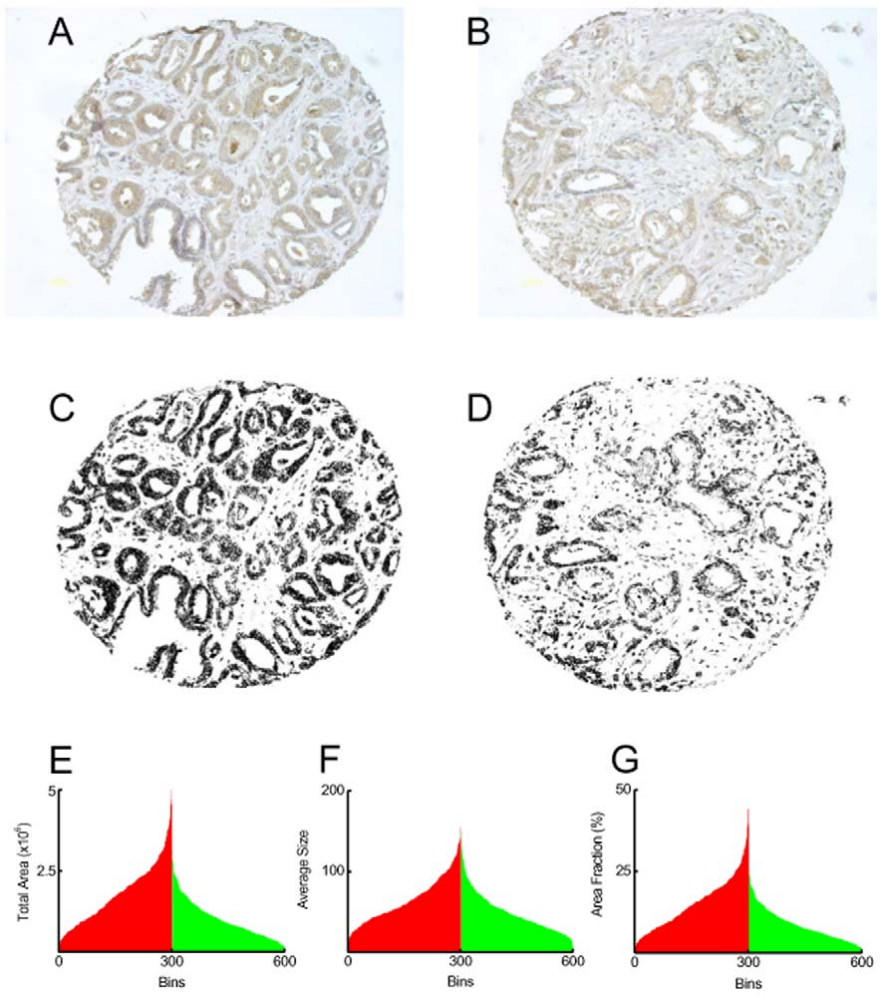
Wnt5A expression in malignant and benign human prostate tissue. Representative malignant (A) and benign (B) tissue core from human prostate arrays used to calculate Wnt5A expression (DAB label, brown, largely in acinar cells). RGB images (A and B) were converted to 16 bit images (C and D) for the quantification of the DAB signal using Analyze Particle protocol in ImageJ software to obtain Total Area stained (E) and Average Size of the particle measured (F), in pixel, and Area Fraction (G, total area divided by the total pixels in the image). Wnt 5A expression was increased in malignant v benign cores (p<0.0001). Each bin is data for an individual, malignant (red, n = 301) or benign (green, n = 299) core.

3,3-diaminobenzidine (DAB) label, representing Wnt5A expression, was observed in both malignant and benign tissue cores (Fig. 1A and B). We quantified the label, in an unbiased manner, by using a reproducible, semi-automated particle analysis (Analyze Particles) protocol using ImageJ software [24] after converting RGB images into 16 bit grayscale (Fig 1C and D) from 600 individual prostate tissue cores (see Materials and Methods). Calculated parameters of count, total area, average size and area fraction are given in Table S2. Integration of area under the curve revealed a 55%, 25% and 45% increase in the total area, average size and area fraction (total area /total pixels), representing the extent and intensity of staining, in malignant cores (n = 301) compared to benign cores (n = 299), respectively (Fig 1E, F and G) that were highly significant (p<0.0001, Student's t-test). These results indicate that Wnt5A expression is increased in malignant compared to benign human prostate.

### Mechanisms of Wnt-signaling in prostate cancer

**Figure 2:**
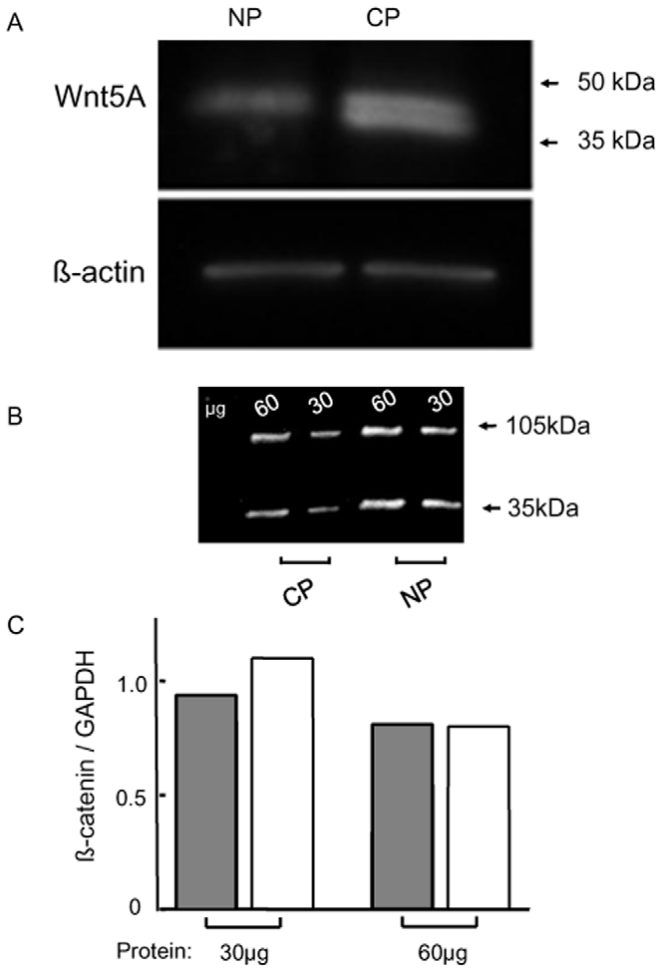
Wnt5A expression in prostate cell lines. Western blot of (A) Wnt5A protein expression, showing higher levels in 1542-CP_3_TX (CP) cells compared to 1542-NPTX (NP) cells. β-actin was used as a loading control. (B) β-catenin (94kDa) expression in cell lysate from 1542-CP_3_TX and 1542-NPTX cell lines (GAPDH, 35kDa, used as a protein loading control on the same blot). (C) Densitometric analysis was performed using commercial software (GeneTools, Synoptics, UK). NP (filled bar)  =  1542-NPTX and CP (hollow bar)  = 1542-CP_3_TX.

Expression of Wnt5A protein was also higher in 1542-CP_3_TX cancer cells compared to matched normal 1542-NPTX cells (Fig. 2A). There was also a concomitant decrease (2.5 fold) in mRNA transcript of CTNNB1 and other elements of Wnt/β-catenin signaling (e.g. axin, DVL1, GSK3β) in cancer cells compared to normal cells (Table S1). Antagonism of β-catenin due to overexpression of Wnt5A, leading to downregulation of Wnt/β-catenin target has been proposed previously in melanoma [25] and Xenopus embryos [11]. β-catenin protein expression, using Western blotting showed a single band (∼94kDa) in a protein concentration dependent manner, in cell lysates from both 1542-CP_3_TX and 1542-NPTX cells (Fig. 2B). Densitometric analysis showed that there was no change in β-catenin protein in the cancer compared to normal cell line (Fig. 2C). Furthermore, according to our oligoarray data [23] a number of Wnt/β-catenin mediated TCF transcription target genes showed decreased mRNA expression in cancer cells (Table S3). A detailed examination at protein and functional level of selected targets of TCF/LEF transcription (e.g. MMP2, MMP14 and TIMP3) [26] confirmed these observations. mRNA expression for these genes was 3–10 fold lower in cancer cells compared to normal cells (Table S1) supported by indirect immunofluorescence (Fig. S1). MMP-14 activity should reflect the equilibrium between MMP-14 catalytic and TIMP3 regulatory activities. MMP activity was 3±0.06 fold lower in the cancer cells using gelatin zymography (Fig. S2). These results confirmed that numerous targets of Wnt/β-catenin signaling pathway were down-regulated at gene, protein or functional level.

**Figure 3:**
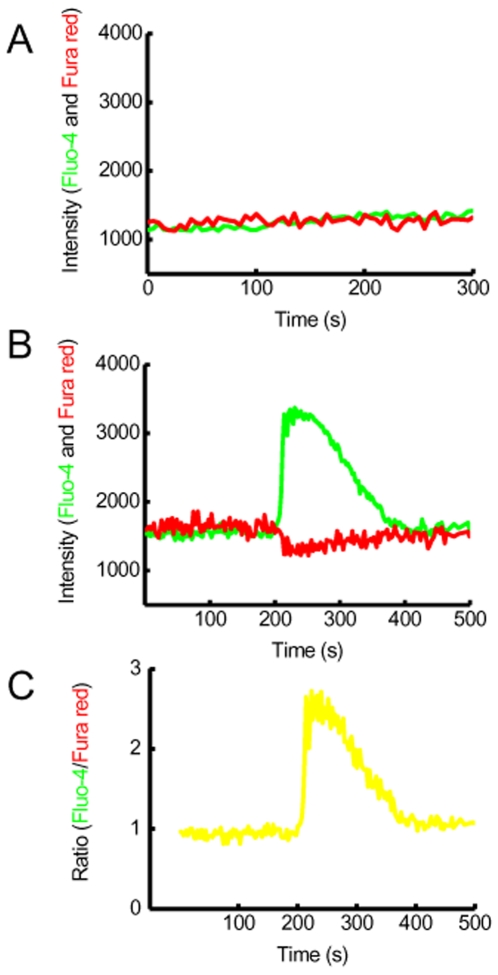
Wnt5A induces calcium release in prostate cancer cells. A representative graph of **c**alcium release in prostate cancer cell line (PC3) as a function of Fluo-4 intensity change over time using confocal live cell imaging. The green line represents the change in the Fluo-4 intensity (green line) in (A) control (after addition of vehicle PBS) or (B) recombinant Wnt5A peptide (100 ng/ml). There was a 3.1±0.1 fold increase in Fluo-4 intensity after addition of Wnt5A (n = 12). In some experiments Fura Red (red line) was loaded with Fluo-4. The ratio of change in Fluo-4 and Fura-red is plotted in (C).

Analysis of gene expression of CTNNB1 and TCF/LEF transcription targets, using Oncomine [27] and GeneSpring software and publicly available microarray datasets for normal (non-neoplastic or benign) *vs* cancer prostate tissue, showed that the expression of almost all Wnt/β-catenin/TCF targets analyzed, except c-myc, was decreased in prostate cancer (Fig. S3). These results are similar to those for prostate cell lines and demonstrate that β-catenin mediated increase in TCF transcription was not likely to be the mechanism of Wnt signaling in prostate cancer. We therefore tested the hypothesis that in the prostate cancer, Wnt signaling is transduced via Wnt/Ca^2+^ pathway. We performed experiments to establish if Wnt5A directly induced calcium release in prostate cells. Addition of Wnt5A peptide induced calcium waves, lasting upto 100s, in prostate cancer cell line with a 3.1±0.1 (n = 12) fold increase in the intensity of Flou-4 from the base line (Fig 3 and Movie S1).

### CaMKII activity and its role in structural plasticity of prostate cells

**Figure 4:**
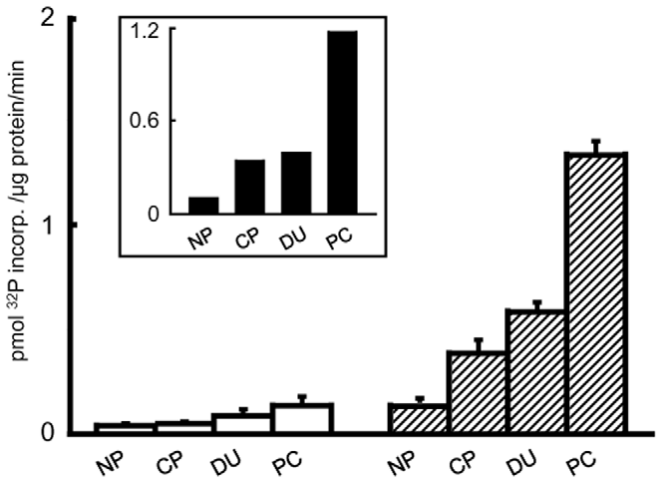
CaMKII activity in prostate cells. The phosphotransferase activity of CaMKII in cell lysates was measured by using a [γ-^32^P] ATP based CaMKII assay (Upstate, UK) with Ca^2+^ (hatched bars) or without Ca^2+^ (hollow bars). *Inset*: Ca^2+^-dependent CaMKII activity was calculated as described in Materials and Methods. Values are mean ± SE from 3 experiments. NP is1542-NPTX, CP is1542-CP_3_TX, DU is DU145 and PC is PC3 prostate cell line.

CamKII is a major transducer of Wnt/Ca^2+^ signaling. In all prostate cell lines CaMKII enzyme activity was Ca^2+^ dependent, least in 1542-NPTX, greater in 1542-CP_3_TX and DU145 and pronounced in PC3 cell line (Fig. 4). There was a 4 and 8 fold increase in the Ca^2+^-dependent CaMKII activity in1542-NPTX and 1542-CP_3_TX cells (Fig. 4), respectively. More importantly, the Ca^2+^ dependent activity of CaMKII was increased by ∼4 fold in 1542-CP_3_TX compared to 1542-NPTX (*inset* Fig. 4). These results indicate an increase in the activity of CaMKII in cancer cells compared to normal cells.

**Figure 5:**
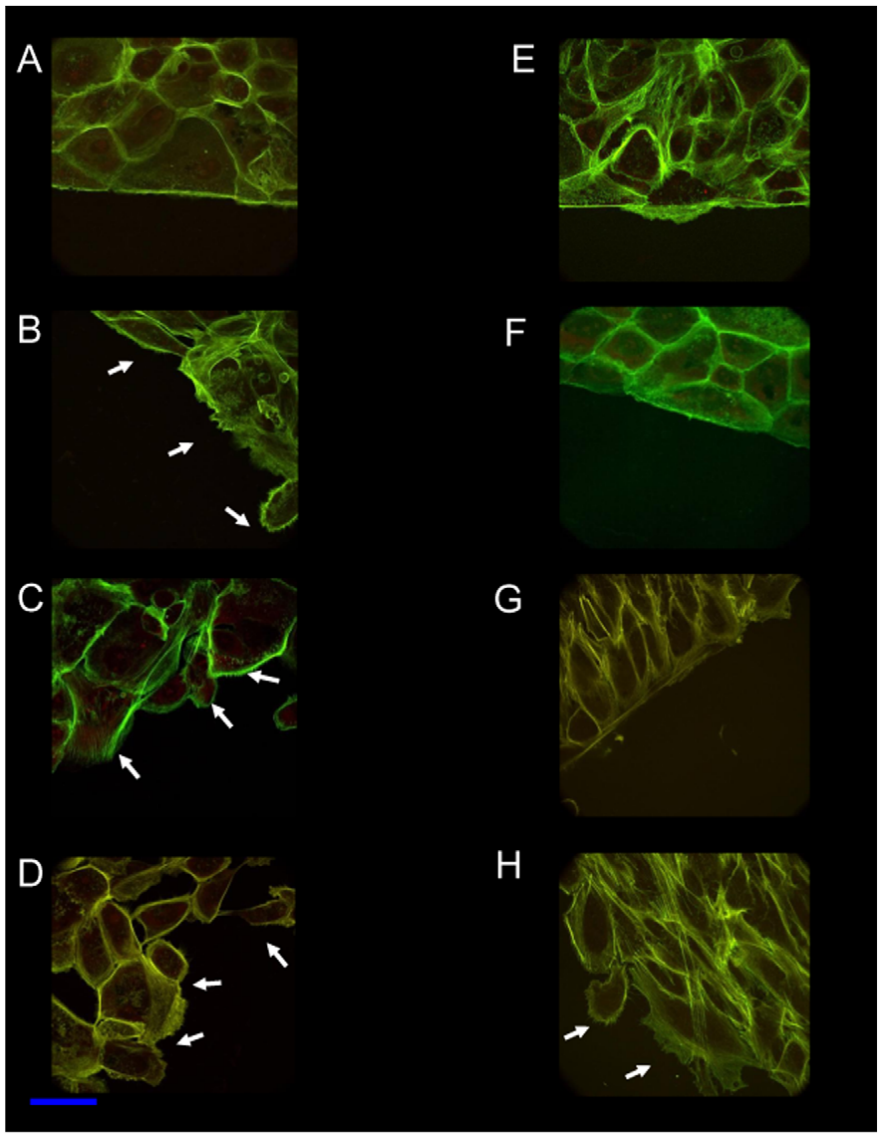
Confocal microscopy of wound edges in prostate cells. Cells were wounded and stained with phalloidin- fluorescein-5-isothiocyanate (FITC,green) to visualize actin filaments using a Leica SP2 confocal microscope. A, B and G are untreated 1542-CP_3_TX, 1542-NPTX and PC3 cells, respectively. C, D and H are AIP (10 µM) treated 1542-CP_3_TX, 1542-NPTX and PC3 cells, respectively. E and F are 1542-CP_3_TX and 1542-NPTX treated with recombinant Wnt5A (100 ng/ml). Irregular wound leading edge and loose of intercellular connections and fine filaments of actin (filopodia like protrusions), white arrows, are visible in 1542-NPTX normal cells and after AIP treatment in 1542-CP_3_TX and PC3 cancer cells. Propidium iodide was used to stain nucleic acid (red). Scale bar = 10 µm

To investigate the role of Wnt signaling in actin cytoskeleton of normal and cancer prostate cells, we used a wound/scratch assay in combination with confocal and scanning electron microscopy and live cell imaging. Firstly, the leading edge of the wound was observed for actin-remodeling, using confocal microscopy after staining with fluorescently labelled phalloidin. In 1542-CP_3_TX cells the leading edge of the wound, at 4 h post-wounding, showed smooth, regular actin staining, with cells appearing in a lamellipodia like formation (Fig. 5A). In 1542-NPTX cells the leading edge of the wound was irregular with morphology of individual cells, some with fine filopodia like structures visible at 4 h (Fig. 5B).

We next tested the following hypotheses: (i) inhibition of CaMKII should disrupt the wound leading edge in prostate cancer cell lines (ii) activation of Wnt5A signaling in 1542-NPTX cells should promote actin remodeling of the wound as observed in 1542-CP_3_TX cells. We used myristoylated autocamtide-2-related inhibitory peptide (AIP), an inhibitor of CaMKII, and recombinant Wnt5A protein (to activate Wnt signaling) in normal and cancer cells to test these hypotheses (Fig. 5). Confocal microscopy of wounded/scratched monolayer of 1542-CP_3_TX cells incubated with AIP (10 µM) displayed disrupted, irregular wound leading edge with fine filopodia (Fig. 5C, arrows) compared to regular wound edge in untreated cells (Fig. 5A). The leading edge of wounded 1542-NPTX cells with or without AIP showed an irregular edge, with loose cell to cell contact and fine actin filament protrusions (Fig. 5B and D). These micrographs indicate that inhibition of CaMKII in 1542-CP_3_TX cancer cells induce filopodia like protrusions. Conversely, wounded 1542-NPTX normal cells, incubated with recombinant Wnt5A protein (100 ng/ml), displayed a regular leading edge (Fig. 5E) of the wound compared to the untreated control (Fig. 5B). No apparent difference was observed in the leading wound edge for untreated vs Wnt5A protein incubated 1542-CP_3_TX cells (Fig. 5A and F).

To validate that actin remodeling was mediated by CaMKII and not via other kinases (e.g. CaMKIV, PKA, PKC, Raf or MAPK1, JNK1α1, or Raf), we used tatCN21a, a specific inhibitor of CamKII [28]. 1542-CP_3_TX cells treated with 5 µM of tatCN21a showed irregular wound edges, loose cell to cell contact and filopodia formation (Fig. S4) as that observed with AIP (Fig. 5). Inhibition of CaMKII also induced, irregular wound edge, loosening of cell to cell contact and filopodia in other prostate cancer cell lines including PC3 (Fig. 5G and H and Fig. S5), DU145 (Fig. S6) and androgen sensitive LnCaP cell line (Fig. S7).

**Figure 6:**
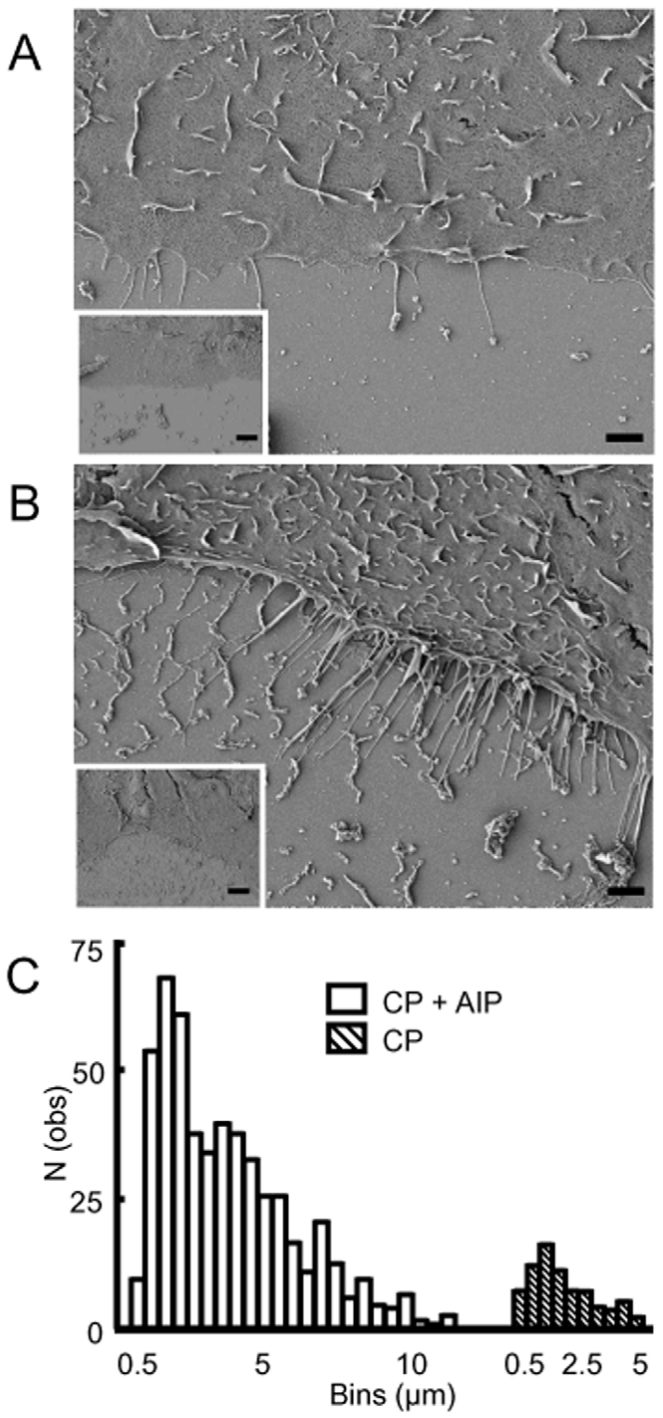
Scanning electron microscopy of the wound edge in 1542-CP_3_TX cells. Main pictures are representative images (scale bar 1 µm) of untreated (A) and AIP (10 µM) treated (B) cells. Inset is the low magnification image (scale bar 10 µm) of the main picture. Irregular wound edge and fine filopodia like protrusion are visible after treatment with AIP (B, main picture). Length of filopodia like protrusions was measured using Image J software and converted to distribution histogram (C) for AIP treated (hollow bars) and untreated (hatched bars) cells. An 8-fold increase in the area under the curve was observed for treated compared to untreated cells using a Gaussian fit.

The mechanism by which CaMKII inhibition caused filopodia formation in prostate cancer cells was next considered. We used scanning electron microscopy to quantify the filopodia like structures (Fig. 6). Low magnification scanning electron micrographs of 1542-CP_3_TX cells show regular and irregular wound leading edges in untreated (*inset* Fig. 6A) and AIP treated cells (*inset* Fig. 6B). High magnification images clearly show numerous and extended fine filopodia like projections from the cell membrane (Fig. 6B) in AIP treated 1542-CP_3_TX cells compared to untreated cells (Fig. 6A). Inhibition of CaMKII with AIP caused a similar effect in PC3 cell line (Fig. 5H and Fig. S5B). The frequency and length of the filopodia was measured manually using ImageJ software. A Gaussian fit of the length distribution histogram of treated and untreated 1542-CP_3_TX cells revealed an 8 fold increase in the length and a 6 fold increase in the overall frequency of filopodia like protrusions in AIP treated compared to untreated 1542-CP_3_TX cells (Fig. 6C and Table 1). The histogram could be fitted for at least two lengths of filopodia like protrusions in 1542-CP_3_TX cells: long (up to 2 µm) and very long (>2 µm). Further analysis showed that there was a 5 fold increase in length but a much greater (15 fold) increase in the frequency of the very long protrusions in AIP treated cells compared to untreated cells (Table 1). These results confirm a major role for CaMKII mediated Wnt signaling in actin remodeling in prostate cancer by decreasing the length and frequency of filopodia.

**Table 1:** Quantification of filopodia like projections in untreated and AIP treated 1542-CP_3_TX cells.

	Wound edge analyzed (µm)	Filopodia (all) / µm	Filopodia (very long) / µm
CP - AIP	716±5.02	0.13±0.02	0.04±0.01
CP + AIP	650±8.41	0.86±0.10*	0.61±0.10^¶^

CP, prostate cancer cells (1542-CP_3_TX) were grown on glass coverslips, scratched/wounded and incubated with (+) or without (−) CaMKII inhibitor (10 µM, AIP) and viewed using a scanning electron microscope. High magnification micrographs were manually analyzed (∼0.7 mm of wound edge) using ImageJ software. Frequency is represented as number of protrusions observed / µm wound length analyzed for all protrusions and a subset population of very long (>2 µm in length) filopodia like protrusions. Both the number and frequency of projections were increased upon treatment with AIP (*^,¶^p<0.001). Data from 2 independent experiments is presented for 10–12 micrographs for each condition manually observed for filopodia like projections along the length of the membrane. Results are means ± SE. Statistical analysis for significance of difference was performed using ANOVA.

### Functional implications of Wnt/Ca^2+^ signaling in prostate cancer cells

**Figure 7:**
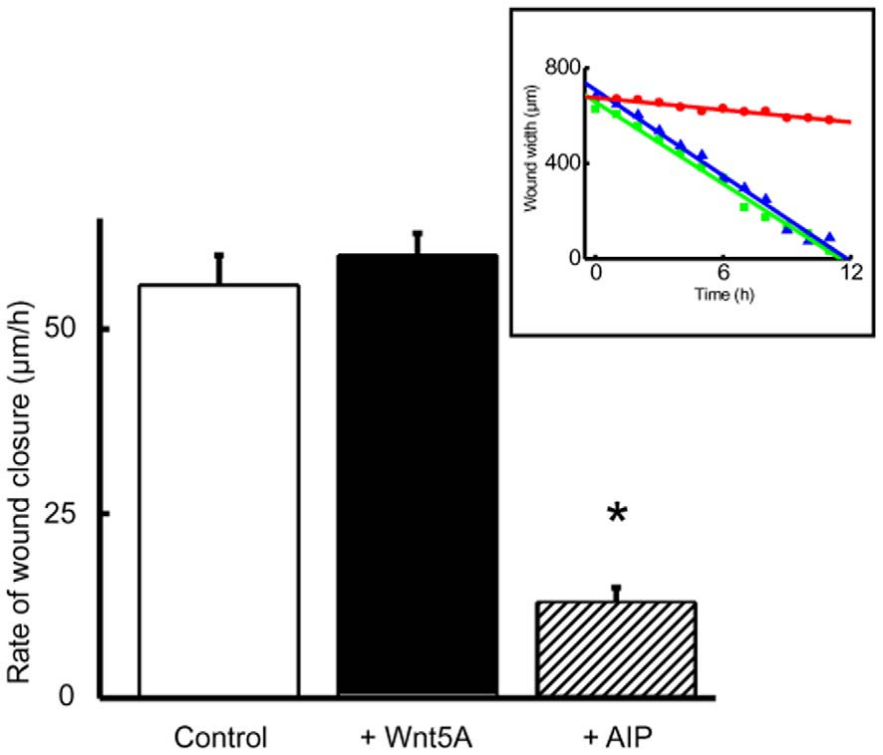
Rate of wound closure and cell motility assay in PC3 cells lines by real-time cell imaging. Confluent PC3 prostate cancer cell lines were wounded and imaged over night at 1h intervals. The images were composited (see Movies S2,control and S3, AIP) and data imported into a spreadsheet. Addition of AIP (10 µM, hatched bar) reduced the rate of wound closure by 80% compared to control (hollow bar, * p<0.001) or Wnt5A (100 ng/ml, solid bar). Data is presented as means ± SE, significance of difference between control and AIP was obtained by ANOVA. Rate of wound closure was determined by performing linear regression to calculate the slope of line (*Inset*, representative of n = 6−7) for untreated control (filled triangles, *R* = 0.99), Wnt5A (filled squares, *R* = 0.99) or AIP (filled circle, *R* = 0.97) treated PC3 cells.

Cytoskeleton plays an integral role in cell motility. To investigate the possible manifestations of cytoskeletal alterations we tested the hypothesis that CaMKII inhibition will decrease cell motility in prostate cancer cell lines. We used the wound scratch assay and live cell imaging with Incucyte (Essen Instruments) to calculate the rate of wound closure as a measure of cell motility. The rate of wound closure was decreased by 80% in PC3 cancer cell lines treated with AIP (Fig. 7 and Movie S2, control and Movie S3, AIP).

## Discussion

Wnt expression and mechanisms of Wnt signaling remains a poorly investigated area in prostate. We demonstrate that (i) Wnt5A protein expression is increased in malignant compared to benign human prostate tissue and in cancer cell lines compared to normal. (ii) Wnt5A is major transducer of Wnt signaling via activation of Wnt/Ca^2+^ pathway and not Wnt/β-catenin pathway in prostate cells. (iii) Activation of CaMKII is a novel and critical regulator of cytoskeleton in prostate cancer. (iv) CaMKII inhibition significantly decreases cell motility and the capacity of wound healing in prostate cancer cell lines.

An understanding of the molecular basis of prostate cancer, and the role played by signaling networks such as Wnt-pathway, requires not only a comprehensive description of gene and protein expression in human prostate tissue, but also a cell system with which to investigate the mechanisms of signaling and its functional consequences in disease. Our prostate tissue array investigations show that there was a highly significant (p<0.0001) increase in both the total area (extent) and average size of the labelled particles (intensity) and area fraction (Fig 1E, F and G and Table S2). These results support the earlier observations of increase in the expression of Wnt5A gene in prostate cancer due to hypomethylation [23] and also a very recent report by Yamamoto *et a*l [29] that used conventional, non-quantiative, histological examination to assess Wnt5A expression prostate. This data is also in agreement with that obtained for normal (1542-NPTX) and cancer (1542-CP_3_TX) cell lines (Fig 2). Taken together the human tissue and cell line data suggest that Wnt5A protein is expressed in normal (or benign) tissue but its expression is dramatically increased in malignant (cancer) prostate tissue and this change is reflected in the cell lines used in this study.

Almost all known transcription targets activated by the Wnt/β-catenin pathway, e.g. CTNNB1, CCCNDs, MMPs, PITX2, CD44, APCDD1, JUN [30]–[32], remain unchanged or are down-regulated in prostate cancer tissue or cell lines compared to normal tissue or cell line (Fig. S3 and Table S1). These results indicate that the cell line dataset largely reflects the gene expression pattern for TCF/LEF regulated genes in both the cell line and the cancer tissue. Furthermore, there was no increase in protein expression or functional activity of downstream targets of canonical Wnt-signaling (e.g. MMP-14). This suggests that 1542-NPTX and 1542-CP_3_TX and PC3 cell lines may be a useful model to investigate the mechanisms of Wnt signaling in prostate cancer as the integrity of Wnt signaling elements in prostate cancer are preserved in these cell lines at the gene, protein and functional levels.

CaMKII is a key mediator of Wnt/Ca^2+^ signaling although a further subset of the non-canonical Wnt signaling via Wnt5A is proposed to be a β-catenin degradation pathway which does not require activation of CaMKII [33]. A 3 fold increase in free intracellular calcium concentration was observed after addition of Wnt5A in prostate cells (Fig 3 and Movie S1). In addition, we did not see a decrease in β-catenin protein expression and the activity of CaMKII was found to increase in prostate cancer cell lines, indicating that Wnt/Ca^2+^ pathway was likely to be operative via CaMKII in prostate cancer.

The multiple roles of CamKII in actin remodelling, cell motility and migration of normal keratinocytes, muscle and nerve cells are known [34]–[36]. However the involvement of CaMKII mediated Wnt/Ca^2+^ signaling in disease has been less well characterized. Pukrop *et al* [37] suggested that the non-canonical pathway may be involved in the increased migration/invasiveness of MCF7 breast cancer cell lines when recombinant Wnt5A is added to the cell culture. However, the mechanisms or whether Wnt5A mediated its effect through CaMKII activation, were not investigated. Cell motility requires two main types of actin based structures in the cell membrane, lamellipodia and filopodia. It has been proposed, in fibroblasts, that filopodia formation from lamellipodia is a tightly regulated process which is partly controlled by actin binding proteins such as enabled (Ena) / vasodilator-stimulated phosphoprotein (VASP) capping the actin filament [38]. Fibroblasts move by extending lamellipodia at the leading edge and formation of fine actin structures at the leading edge causes retrograde movement [39]; sequestration of Ena/VASP to mitochondria increases whereas its targeting to cell membrane reduces motility [38]. The role of Wnt5A mediated signaling in cell motility [37] and other intermediary proteins (e.g. Ena/VASP, Arp2/3) in filopodia formation has been investigated in human [14], [40] and mouse [41] melanoma cells. Weeraratna and colleagues [40] suggested that Wnt5A increased cell motility in human melanoma cell lines by activation of protein kinase C (PKC). Witze *et al* [14] showed that acute response of melanoma cell lines to Wnt5A involves recruitment of major cytoskeletal proteins (actin and myoxin IIB) and Frizzled 3 and melanoma cell adhesion molecule into an intracellular structure via Wnt5A regulation of Rab4 and RhoB guanosine triphosphatases. Another study investigated the role for Ror2 kinase in Wnt5A induced cell migration [42]. Unlike the data presented here, these studies [40], [42] neither investigated nor directly manipulated CaMKII activity in their experiments or investigated its role in filopodia formation. We used tatCN21a, a specific inhibitor of CaMKII (but not CaMKIV, PKA, PKC, MAPK1, JNK1α1, or Raf). Treatment of prostate cancer cells with tatCN21a also caused a loosening of cell to cell contact and induced filopodia formation (Fig. S4) similar to the results obtained with AIP (Fig. 5). These results indicate that direct inhibition of CaMKII (and not CaMKIV, PKC, PKA and other kinases) results in the loss of cell to cell contact and filopodia formation in prostate cancer cells.

In prostate cancer cell lines (1542-CP_3_TX and PC3) a smooth, regular wound edge is observed with close cell to cell contact preceding the lamellipodia like leading edge (Figs. 4 and 5). In contrast, treatment with AIP induces an increase in filopodia like protrusions with a loosening of cell to cell contact preceding the wound edge (Fig. 5 and 6 and Table 1). Indeed the rate of wound closure was decreased by 80% after inhibition of CaMKII in cancer cell line (PC3). Conversely, addition of Wnt5A to normal cell line (1542-NPTX) increased the rate of wound closure (µm/h) compared to untreated cells by 25±4%. We suggest that activation of CaMKII via Wnt5A signaling is advantageous to cancer cell mobility as it suppresses formation of fine filopodia that induce retrograde movement thus reducing cell mobility.

Protein expression of Wnt5A in human prostate cancer and mechanisms of Wnt signaling in normal and cancer prostate cell lines, described here, provide the first framework within which the exact participation of Wnt receptors and secretory frizzled related proteins [20], [21] and various actin binding proteins and intermediary signaling molecules such as Ena/VASP and ERK1 [34], [38], could be investigated in cancer. Further investigations to identify the receptor(s) for Wnt5A binding and other proteins involved in the downstream signaling via CaMKII in cell motility in prostate cancer will help to elucidate the novel role of CaMKII in the suppression of filopodia formation to increase cell motility in prostate cancer. In conclusion, from the results presented here and our recent study [23] showing hypomethylation as a regulator of Wnt5A gene transcription in prostate, the following sequence could be envisaged: (i) Gene expression of Wnt5A is increased in cancer compared to normal cells due to hypomethylation of the gene promoter region (ii) Increase in gene expression results in increased protein expression of Wnt5A in human prostate cancer (iii) Wnt/Ca^2+^ pathway (and not β-catenin pathway) is activated in prostate cancer cells (iv) Signaling via Wnt/Ca^2+^ pathway activates CaMKII (v) CaMKII activity, most likely via intermediary signaling involving actin binding proteins, causes a major reorganization of cytoskeleton in cancer cells by decreasing the length and frequency of fine, filopodia like actin structures. (vi) Cytoskeletal remodeling due to CaMKII causes an increase in cell motility. Targeting of Wnt/Ca^2+^ signaling, particularly CaMKII may provide a useful tool in prostate cancer therapy.

## Materials and Methods

### Cell culture

The human prostate epithelial cell line1542-NPTX and prostate cancer cell line 1542-CP_3_TX [43] (derived from normal and prostate cancer tissue from the same patient [43]) were obtained from S L Topalian, National Cancer Institute, NIH, USA, originators of these cell lines. Prostate carcinoma cell line PC3 was obtained from M E Kaighn the originators of this cell line [44]. DU145 was obtained from the cell bank maintained by Jørgen Fogh at the Sloan Kettering Cancer Center, NY, USA, from a deposit made by the originator of this cell line [45]. PC3 and DU145 were cultured in RPMI 1640 (Invitrogen) medium containing 5 mM L-glutamine and fetal bovine serum (FBS) and 1542-NPTX and 1542-CP_3_TX were maintained in Keratinocyte-SFM (KSFM) medium and supplements (Invitrogen) with 5% FBS and KSFM supplements (Invitrogen).

### Quantitative real time PCR

Fluorescent real-time PCR (TaqMan, Applied Biosystems, UK) was used to verify gene expression in 1542-NPTX and 1542-CP_3_TX originally identified from the microarray experiments [23]. RNA isolation is described elsewhere [23]. TaqMan probes and primers for real-time PCR were purchased as a pre-developed assay system; Applied Biosystems assay IDs for the probes used and a brief protocol is given in Table S4. Each sample was tested in quadruplicate and results were analyzed using sequence detector software (SDS v2.2 – Applied Biosystems, UK). Relative quantitation was performed using the 2^−ΔΔ^C*_T_* method [46].

### Use of human tissue arrays for Wnt5A expression in prostate tissue

#### Ethics approval

Ethical approval was given by the Joint UCL/UCLH committees on the ethics of human research. The review board approved the use of human tissue for prostate cancer research, in compliance with the International Committee on Harmonisation of Good Clinical Practice (ICH GCP). Radical prostatectomy surgical samples, all but fivefrom the period of July 1994-April 2002, were used to construct tissue arrays (one malignant and four benign samples from December 1991 are also included in th analysis). The samples were anonymized during the construction of the tissue arrays and used for various immunohistochemical studies in a project approved by the UCL/UCLH ethics committee. Details of patient selection, disease state and construction details of tissue blocks are given elsewhere [47].

### Measurement and analysis of Wnt5A expression in prostate tissue arrays

Patient selection, disease state and construction details of tissue blocks are given elsewhere [47]. Briefly, tissue blocks were constructed using archival formalin-fixed, paraffin-embedded radical prostatectomy specimens from the 82 patients with pathological state of pT3a or b and pre-operative PSA stage of >3. All radical prostatectomy specimens were examined by a urological pathologist. 3 µm sections were cut from the tissue arrays onto coated slides and dried overnight at 60°C, prior to performing standard antigen retrieval. Immunostaining was performed using standard 3,3-diaminobenzidine staining protocol [47] and 2 µg/ml of Wnt5A primary antibody (clone AF645, R&D Systems) on an automated Bond maX™ machine (Vision BioSystems) using the Bond polymer detection system kit (containing post primary antibodies), according to manufacturers protocol, at high contrast (DS9173).

Image of each core was acquired with a Nikon DXM 1200 digital imaging system (image resolution = 3840×3072 pixels) attached to a Nikon Diaphot (20x magnification) at standardized bright-field settings. A reproducible, automated method was employed to quantify the DAB signal on benign and malignant human prostate tissue cores using ImageJ software [24]. Macros were written to execute the following sequence of events for acquired jpeg images: 1. Open image 2. Convert to 16 bit image 3. Set threshold (152,195) 4. Analyze particle (Size 0.5- Infinity, Circularity 0.00–1.00). Save image 6. Save particle information (count, total area, average size and area fraction) into an excel spreadsheet (*rsb.info.nih.gov/ij/docs/pdfs/examples.pdf).* Units are default ImageJ setting (pixels). Wnt5A expression was observed to be largely epithelial (see Figure 1); set threshold parameters were chosen after manual analysis of random cores to quantify this signal. A contiguous spreadsheet for all the usable cores (n =  301 malignant cores and n = 299 benign cores) was constructed and statistical analysis using Student's t-test was performed.

### Intracellular calcium imaging

PC3 cells were grown as a monolayer in 35 mm FluoroDish (WPI). Cell growth medium was replaced with 1 ml PBS prior to loading with calcium indicators Fluo-4 (Invitrogen) alone or in conjunction with Fura Red (Invitrogen) at 1 ng/ml for 1 h at 37°C. Live confocal imaging was performed using an Olympus FluoView FV 1000 confocal microscope equipped with a 20x dry objective (numerical aperture = 0.75). Calcium indicators were excited with an argon laser line (488 nm) and emissions recorded in the green channel (510–580 nm) for Fluo-4 and red channel (600–700 nm) for Fura Red after addition of vehicle control (PBS) or Wnt5A (100 ng/ml). Image and data acquisition was performed using FluoView 1000 software (Olympus). Fluorescent intensity was measured and data exported as a tab delimited file for further analysis using Image J and Origin (Microcal) software. Experiments were repeated in 3 different passages of cell line.

### CaMKII assay

The phosphotransferase activity of CaMKII in cell lysates was measured using the CaMKII assay kit (Upstate, UK) according to manufacturer's protocols. The assay is based on phosphorylation of specific substrate peptide (KKALRRQETVDAL) by the transfer of the γ-phosphate of adenosine-5′-[γ-^32^P]triphosphate ([γ-^32^P]ATP) by CaMKII.

The Ca^2+^ dependent CaMKII activity is calculated as a sum of ser/thr kinase activity in the presence of PKA/PKC inhibitors with or without Ca^2+^.

### Peptides and inhibitors

CaMKII inhibitors myristoylated autocamtide-2-related inhibitory peptide (AIP) (Biomol, UK) and tatCN21a (a specific inhibitor of CaMKII [28]) was purchased from the University of Colorado, USA. Recombinant Wnt5A protein (R&D Systems, USA) was used to activate Wnt signaling.

### Wound/scratch assay and microscopy of prostate cell lines

Cell lines (1542-NPTX, 1542-CP_3_TX, PC3 and DU145) were grown on glass coverslips to confluency and scratched using a plastic pipette tip. Details of fixing, staining and microscopy are given in Methods S1. Rate of wound closure, as a measure of cell motility, was determined by live cell imaging using a specialized WoundMaker, ImageLock plates and Incucyte (Essen Instruments). Wound width was photographed for 12 h, images were composited (Movies S2, control and S3, AIP) and data saved into an excel spreadsheet for analysis. Details are provided in Methods S1. Statistical analysis for significance of difference was performed using ANOVA. For scanning electron microscopy (JSM-7410 scanning electron microscope, Jeol, Japan), cells from two independent preparations were used visualized using. Detailed methodology is given in Methods S1.

### Gene expression data analysis

We used Microarray Suite 5.0 analysis software (as described previously, [23], GeneSpring (Agilent, UK) analysis software and Oncomine (www.oncomine.org, [27] for the analysis of cell lines and publicly available prostate microarray datasets.

## Supporting Information

Figure S1 MMP-14 and TIMP3 expression in prostate cell lines. MMP-14 protein expression (green) was detected at a higher level in normal 1542-NPTX cells (A) compared to cancer 1542-CP_3_TX cell line (B); scale bar  =  10μm. TIMP3 protein expression (green) was also greater in 1542-NPTX cells (C) compared to 1542-CP_3_TX cancer cells (D); scale bar  =  5μm. DAPI (blue) was used to visualize the cell nuclei.
(0.83 MB TIF)

Figure S2 Gelatin zymography of the activated MMP2 (62kDa protein band), shows a higher level of activated MMP2 in the MT1-MMP in normal 1542-NPTX (NP) compared to cancer 1542-CP_3_TX (CP) cell lines. HT1080 was used as a positive control for the activity of MT1-MMP complex (50). A representative gel of 3 independent experiments is shown.
(0.25 MB TIF)

Figure S3 Gene expression analysis of targets of TCF (the nuclear mediator of Wnt/beta-catenin signaling) transcription are downregulated in prostate cancer (A to G). Gene expression analysis of targets of TCF: (A) APCDD1, (B) CCND1 (C) CD44, (D) Jun, (E) Myc (F) CTNNB1 and (G) WNT5A. Box plots have been reproduced from www.oncomine.org (see Rhodes et al, 2004), using a P-value threshold of 0.01-0.0001 for prostate cancer v normal (non-neoplastic, normal, normal adjacent or benign prostatic hyperplasia) tissue studies* only. Blue box  =  normal, red box  =  cancer. All targets analyzed in cancer tissue, except c-myc, were down-regulated in cancer compared to normal tissue. These data are similar to those observed in 1542-NPTX v 1542-CP_3_TX cell lines (derived from tissue with Gleason score 6-8, Bright et al 1997). Two other TCF targets, namely PITX2 and PLAU, did not show a significant change in expression between cancer v normal analysis of Yu et al, 2004 and Lapointe et al, 2004, respectively. 9 different microarray studies were used for this analysis (references appear under each box plot). The original plots with sub-classes can be obtained from oncomine.org.
(0.06 MB PDF)

Figure S4 Confocal microscopy of 1542-CP_3_TX wounded prostate cancer cell line control (A) and tatCN21a (5µM) treated (B). tatCN21a is a CamKII specific inhibitor, which does not inhibit other kinases (e.g., CamKIV. PKA, PKC or Raf and others). Speciific inhibition of Cam KII by tatCN21a induces fine filopodia (arrows) and irregular wound edges compared to a regular wound edge (arrow heads) control wounds. tatCN21a (a peptide that specifically inhibits CamKII and not CamKIV, PKA, PKC, Raf or MAPK1, JNK1α1, or Raf) causes disruption of cell to cell contact and filopodia formation in prostate cancer cells (1542CP_3_TX).
(0.68 MB TIF)

Figure S5 Scanning electron microscopy of the leading wound edge in PC3 (A and B) prostate cancer cell lines with or without AIP treatment (scale bar  =  10μm). Representative images of untreated (A) and AIP treated (B) cells.
(0.44 MB TIF)

Figure S6 Wound scratch assay on DU145 prostate cancer cell line. Immunofluorescence microscopy of wounded DU145 prostate cancer cell line control (A) and AIP treated at 4h. Inhibition of Cam KII by AIP induces irregular wound edges.
(1.10 MB TIF)

Figure S7 Scanning electron microscopy of the leading wound edge in LnCaP prostate cancer cell lines with (A) or without (B) AIP treatment (scale bar  =  10μm). Representative images of untreated (A) and AIP treated (B) cells. Insets C and D are the low magnification (scale bar  =  100μm) images of LnCaP wound edge. These results are similar to those observed for 1542-CP_3_TX prostate cancer cells (Figure 6).
(0.45 MB TIF)

Methods S1 (0.03 MB DOC)

Table S1 mRNA quantitation using real time PCR mRNA and comparative CT method in prostate cell lines. 2-ΔΔCT method was used to calculate relative quantities of mRNA in 1542-CP_3_TX relative to 1542-NPTX, using 18S rRNA as normalization controls. A. The range is determined by evaluating the expression 2-ΔΔCT with ΔΔCT + s and ΔΔCT - s, where s  =  SD of the ΔΔCT value.
(0.03 MB DOC)

Table S2 Quantitation of Wnt5A expression in malignant and benign human prostate tissue using ImageJ software. DAB label, representing Wnt5A expression was quantified in an unbiased manner, by using a reproducible, semi-automated particle analysis (Analyze Particles) protocol with ImageJ software. Over 600 individual prostate tissue cores (see Materials and Methods) RGB images were converted into 16 bit grayscale (e.g., from images shown in Fig 1). The results are mean ± SE for the calculated parameters of count, total area, average size and area fraction.
(0.03 MB DOC)

Table S3 Genes under the transcriptional regulation of Wnt/β-catenin/TCF pathway are downregulated in the prostate cancer cell line. Downstream targets known to be under the control of canonical Wnt-signaling pathway are down-regulated in 1542-CP_3_TX compared to 1542-NPTX cells, possibly due to the activation of the non-canonical Wnt-signalling in these cells. (Other Wnt/β-catenin pathway targets such as PITX2, APCDD1 or JUN did not show a significant change in expression in normal and cancer cell lines.) Oligoarray data from Wang et al, Oncogene, 2007 (doi: 10.1038/sj.onc.1210472).
(0.03 MB DOC)

Table S4 Applied Biosystems probe IDs for TaqMan assay. 18S rRNA (Hs99999901_s1) was used as endogenous controls. Quantitative PCR was performed using ABI Prism 7900 with MicroFluidic cards (Applied Biosystems) according to manufacturer's protocols, with each reaction containing 8ng of reverse transcribed RNA in a 2μl reaction mix. The following cycling parameters were employed: 48oC for 30 min, 95oC for 10 min, followed by 40 cycles of 95oC for 15 sec and 60oC for 15 sec.
(0.03 MB DOC)

Movie S1 A representative time lapse imaging of Wnt5A induced calcium release in PC3 prostate cancer cell line. Left frame (green) for Fluo-4 in the green channel and frame on the right (red) is for Fura red in the red channel.
(315.40 MB AVI)

Movie S2 Cell motility in untreated prostate cell line. Video of wound scratch assay in control, untreated, prostate cancer cell line. Similar videos were used for calculating the rate of wound closure (Fig 7).
(7.88 MB AVI)

Movie S3 Cell motility in AIP treated prostate cell line. Video of wound scratch assay of prostate cancer cell line + AIP an inhibitor of CamKII. Similar videos were used for calculating the rate of wound closure (Fig 7).
(7.20 MB AVI)
