# Supplementary figures and images for: A Novel Role for Wnt/Ca2+ Signaling in Actin Cytoskeleton Remodeling and Cell Motility in Prostate Cancer
Source: PLoS One. 2010 May 4;5(5):e10456. doi: 10.1371/journal.pone.0010456 (PMC2864254; doi:10.1371/journal.pone.0010456)

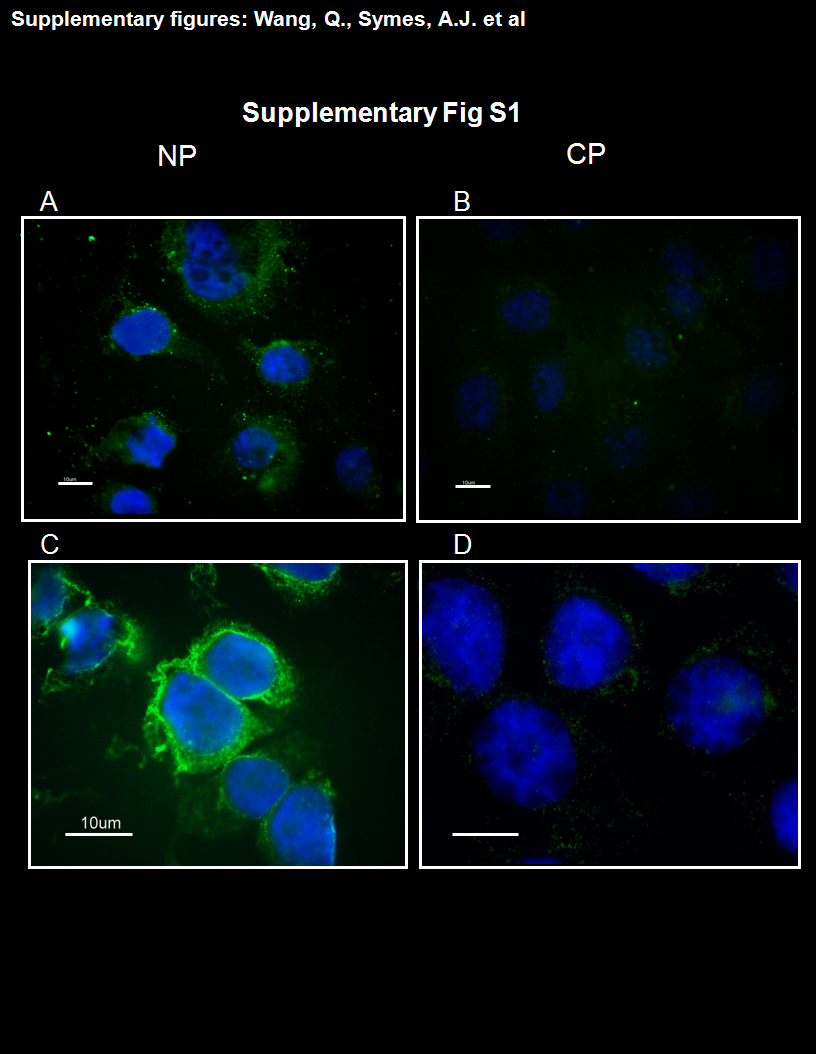

Supplement: Figure S1 — MMP-14 and TIMP3 expression in prostate cell lines. MMP-14 protein expression (green) was detected at a higher level in normal 1542-NPTX cells (A) compared to cancer 1542-CP3TX cell line (B); scale bar = 10μm. TIMP3 protein expression (green) was also greater in 1542-NPTX cells (C) compared to 1542-CP3TX cancer cells (D); scale bar = 5μm. DAPI (blue) was used to visualize the cell nuclei. (0.83 MB TIF) [file pone.0010456.s001.tif]

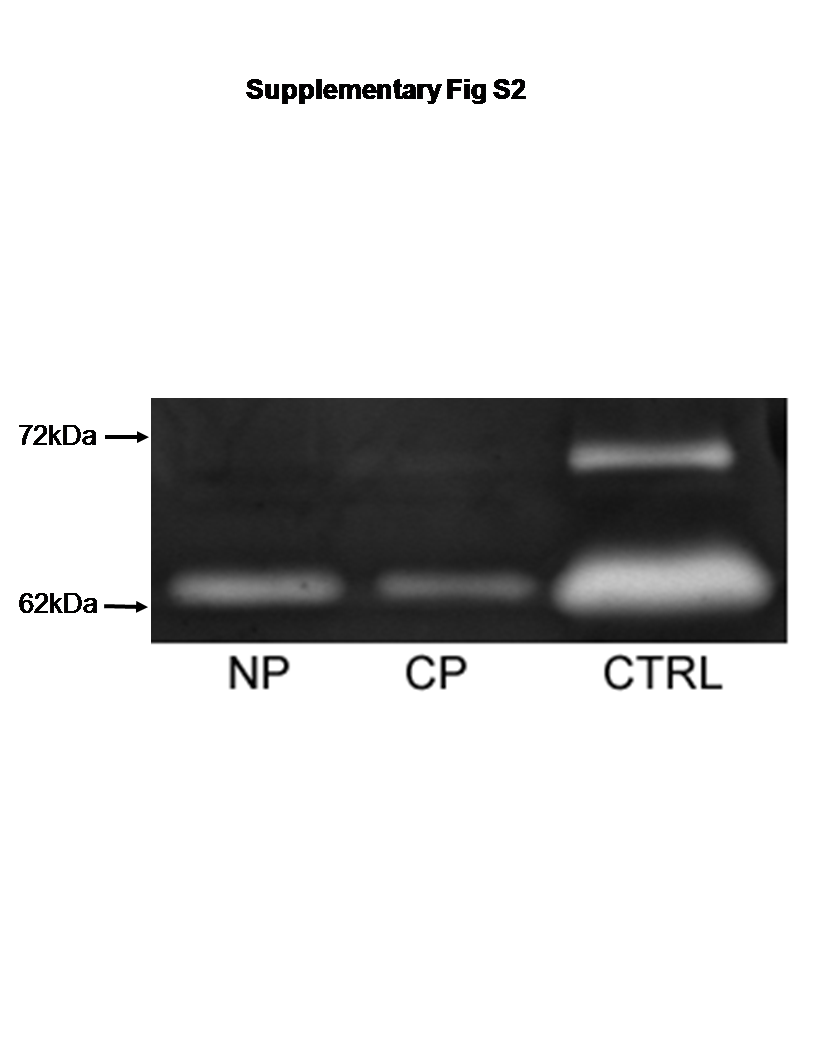

Supplement: Figure S2 — Gelatin zymography of the activated MMP2 (62kDa protein band), shows a higher level of activated MMP2 in the MT1-MMP in normal 1542-NPTX (NP) compared to cancer 1542-CP3TX (CP) cell lines. HT1080 was used as a positive control for the activity of MT1-MMP complex (50). A representative gel of 3 independent experiments is shown. (0.25 MB TIF) [file pone.0010456.s002.tif]

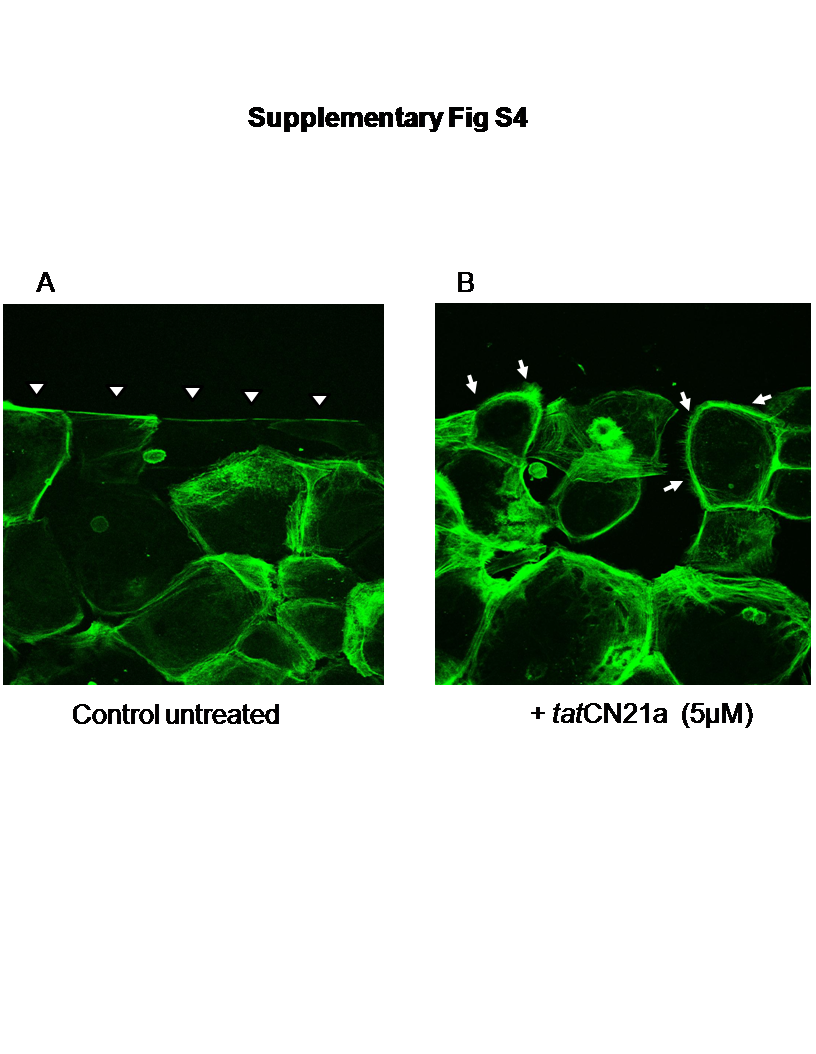

Supplement: Figure S4 — Confocal microscopy of 1542-CP3TX wounded prostate cancer cell line control (A) and tatCN21a (5µM) treated (B). tatCN21a is a CamKII specific inhibitor, which does not inhibit other kinases (e.g., CamKIV. PKA, PKC or Raf and others). Speciific inhibition of Cam KII by tatCN21a induces fine filopodia (arrows) and irregular wound edges compared to a regular wound edge (arrow heads) control wounds. tatCN21a (a peptide that specifically inhibits CamKII and not CamKIV, PKA, PKC, Raf or MAPK1, JNK1α1, or Raf) causes disruption of cell to cell contact and filopodia formation in prostate cancer cells (1542CP3TX). (0.68 MB TIF) [file pone.0010456.s004.tif]

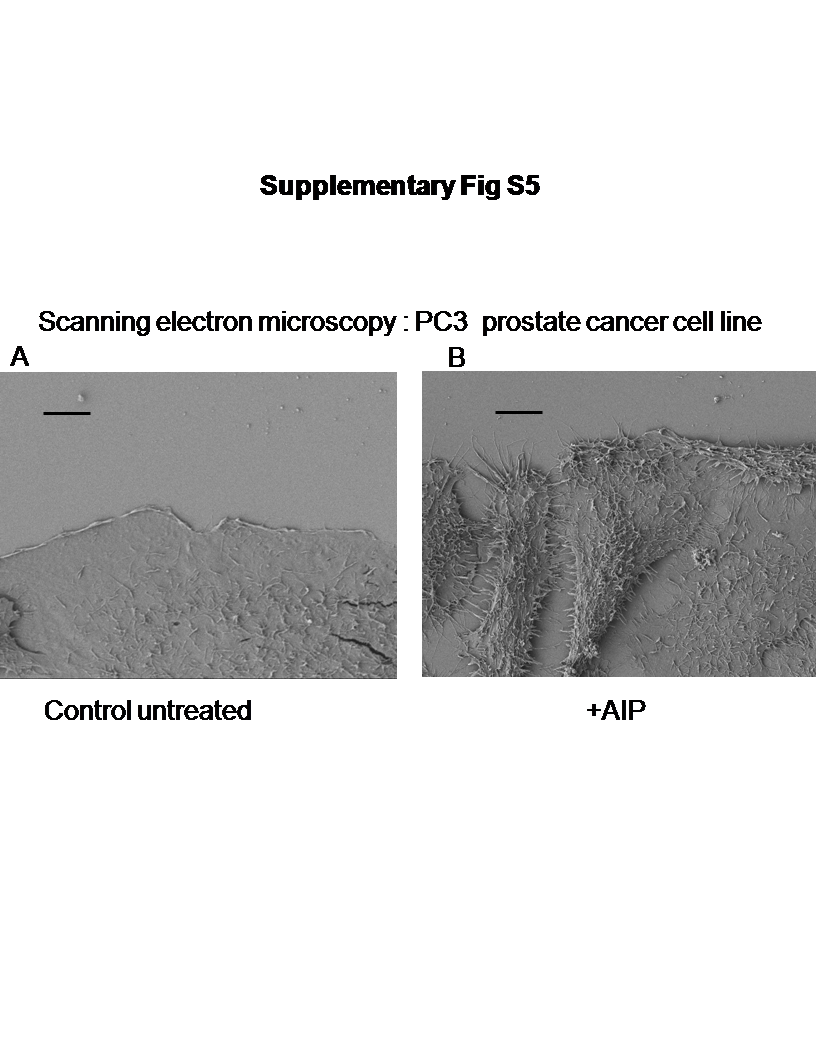

Supplement: Figure S5 — Scanning electron microscopy of the leading wound edge in PC3 (A and B) prostate cancer cell lines with or without AIP treatment (scale bar = 10μm). Representative images of untreated (A) and AIP treated (B) cells. (0.44 MB TIF) [file pone.0010456.s005.tif]

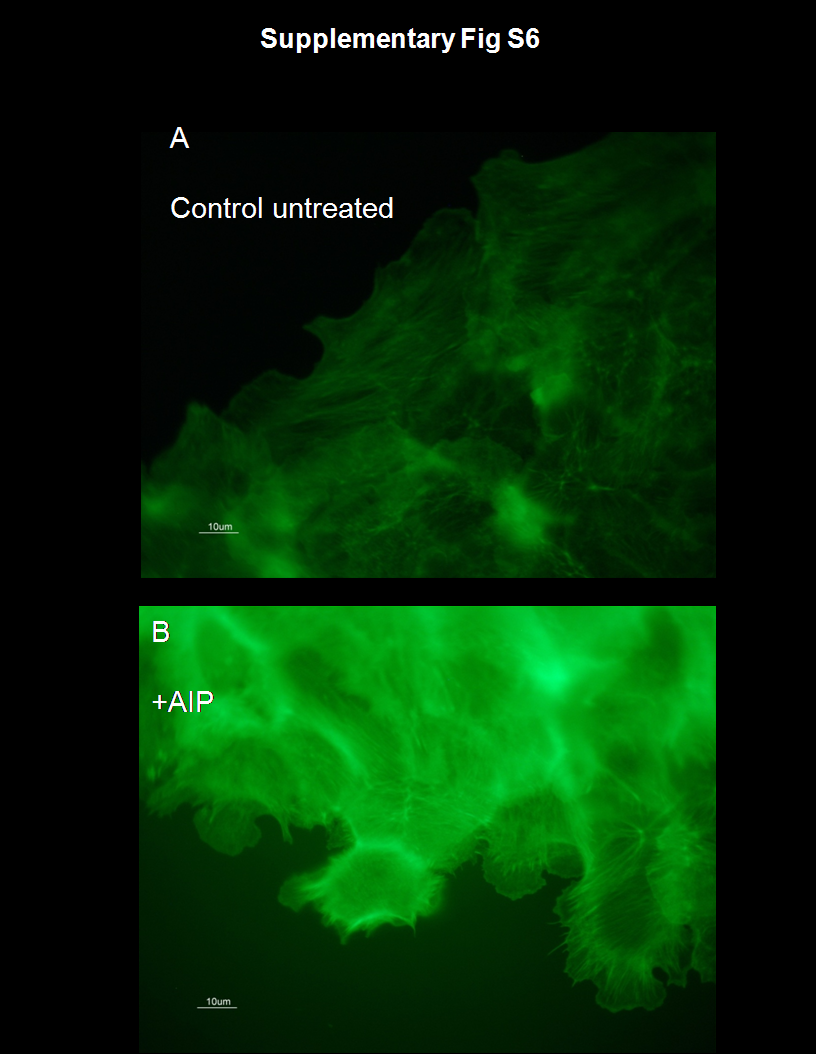

Supplement: Figure S6 — Wound scratch assay on DU145 prostate cancer cell line. Immunofluorescence microscopy of wounded DU145 prostate cancer cell line control (A) and AIP treated at 4h. Inhibition of Cam KII by AIP induces irregular wound edges. (1.10 MB TIF) [file pone.0010456.s006.tif]

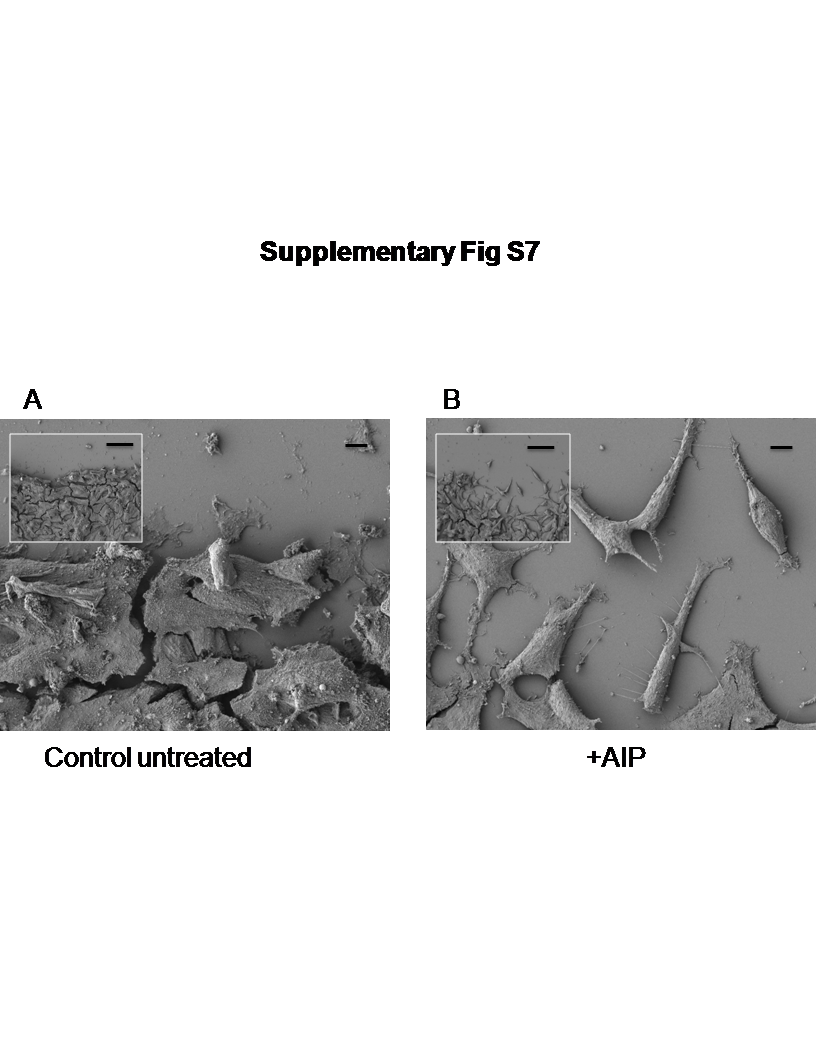

Supplement: Figure S7 — Scanning electron microscopy of the leading wound edge in LnCaP prostate cancer cell lines with (A) or without (B) AIP treatment (scale bar = 10μm). Representative images of untreated (A) and AIP treated (B) cells. Insets C and D are the low magnification (scale bar = 100μm) images of LnCaP wound edge. These results are similar to those observed for 1542-CP3TX prostate cancer cells (Figure 6). (0.45 MB TIF) [file pone.0010456.s007.tif]
