# Supplementary material for: A Novel Role for Wnt/Ca2+ Signaling in Actin Cytoskeleton Remodeling and Cell Motility in Prostate Cancer
Source: PLoS One. 2010 May 4;5(5):e10456. doi: 10.1371/journal.pone.0010456 (PMC2864254; doi:10.1371/journal.pone.0010456)
Supplement: Figure S3 — Gene expression analysis of targets of TCF (the nuclear mediator of Wnt/beta-catenin signaling) transcription are downregulated in prostate cancer (A to G). Gene expression analysis of targets of TCF: (A) APCDD1, (B) CCND1 (C) CD44, (D) Jun, (E) Myc (F) CTNNB1 and (G) WNT5A. Box plots have been reproduced from www.oncomine.org (see Rhodes et al, 2004), using a P-value threshold of 0.01-0.0001 for prostate cancer v normal (non-neoplastic, normal, normal adjacent or benign prostatic hyperplasia) tissue studies* only. Blue box = normal, red box = cancer. All targets analyzed in cancer tissue, except c-myc, were down-regulated in cancer compared to normal tissue. These data are similar to those observed in 1542-NPTX v 1542-CP3TX cell lines (derived from tissue with Gleason score 6-8, Bright et al 1997). Two other TCF targets, namely PITX2 and PLAU, did not show a significant change in expression between cancer v normal analysis of Yu et al, 2004 and Lapointe et al, 2004, respectively. 9 different microarray studies were used for this analysis (references appear under each box plot). The original plots with sub-classes can be obtained from oncomine.org. (0.06 MB PDF) [file pone.0010456.s003.pdf]

Supplementary Fig S3

APCDD1

Adenomatosis polyposis coli down-regulated 1

A

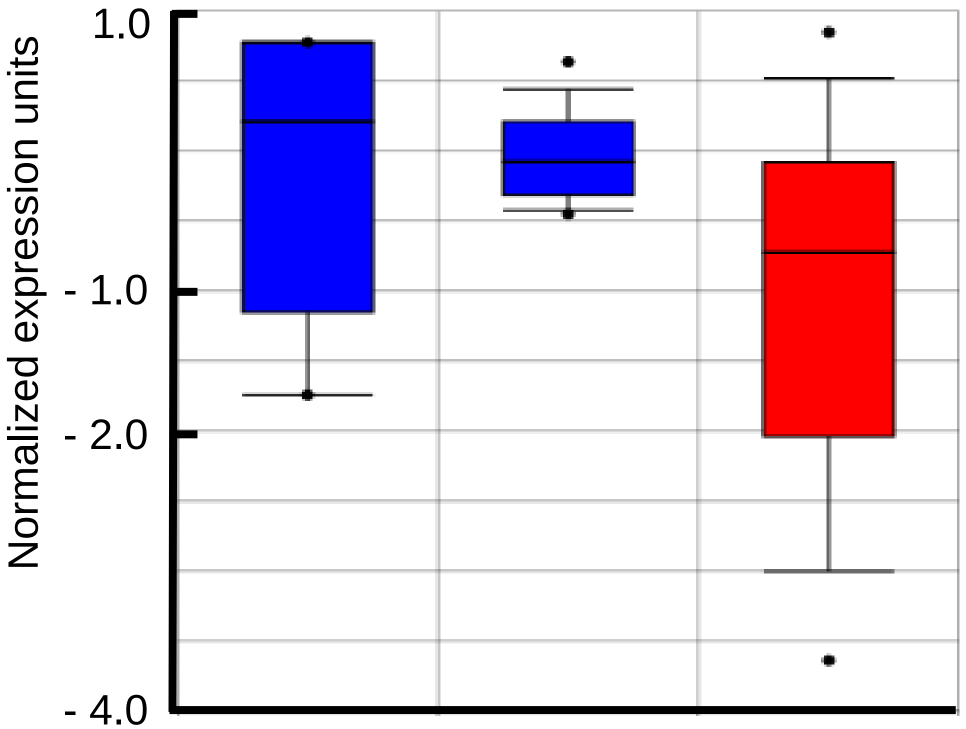

Dhanasekaran SM, Barrette TR, Ghosh D, Shah R, Varambally S *et al.* (2001) *Nature* 412: 822-826.

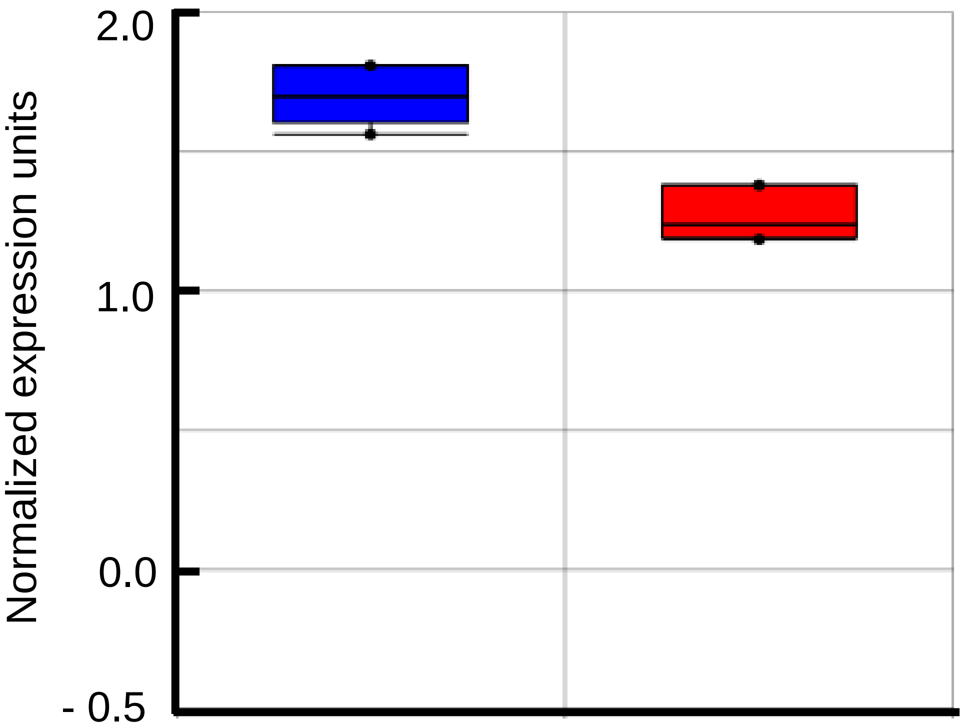

Varambally S, Dhanasekaran SM, Zhou M, Barrette TR, Kumar-Sinha C *et al.* (2002) *Nature* 419: 624-629

CCND1

Cyclin D1

Supplementary Fig S3

B

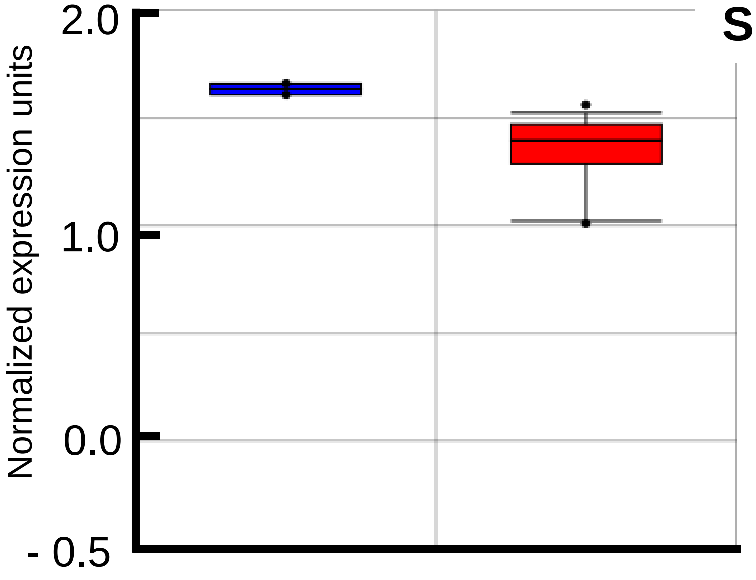

Welsh JB, Sapinoso LM, Su AI, Kern SG, Wang-Rodriguez J *et al.* (2001) *Cancer Res.* 61: 5974-5978.

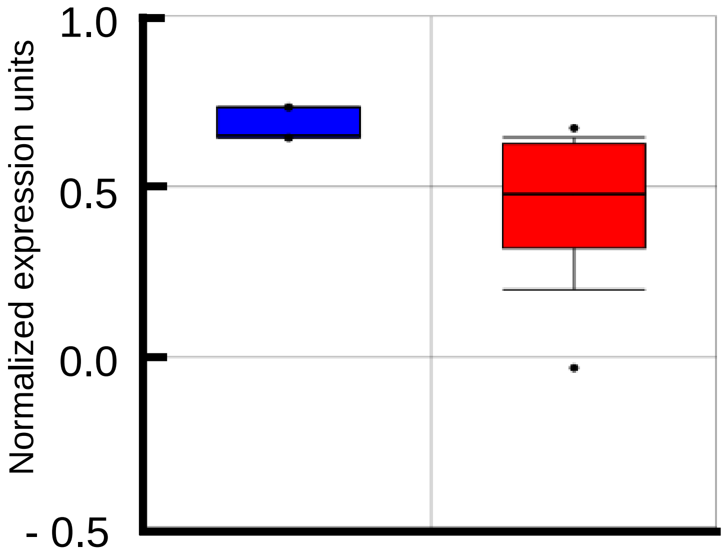

LaTulippe E, Satagopan J, Smith A, Scher H, Scardino P *et al.* (2002) *Cancer Res.* 62: 4499-4506.

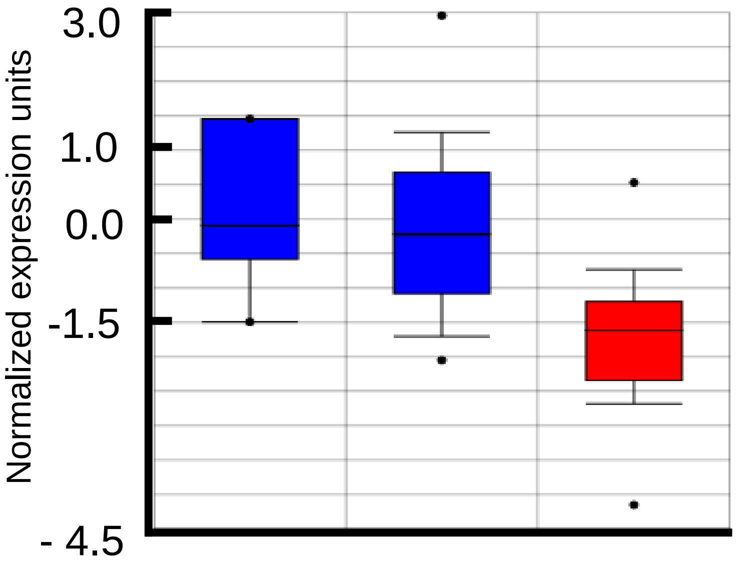

Dhanasekaran SM, Barrette TR, Ghosh D, Shah R, Varambally S *et al.* (2001) *Nature* 412: 822-826.

CD44

CD-44 molecule (Indian blood group)

Supplementary Fig S3

C

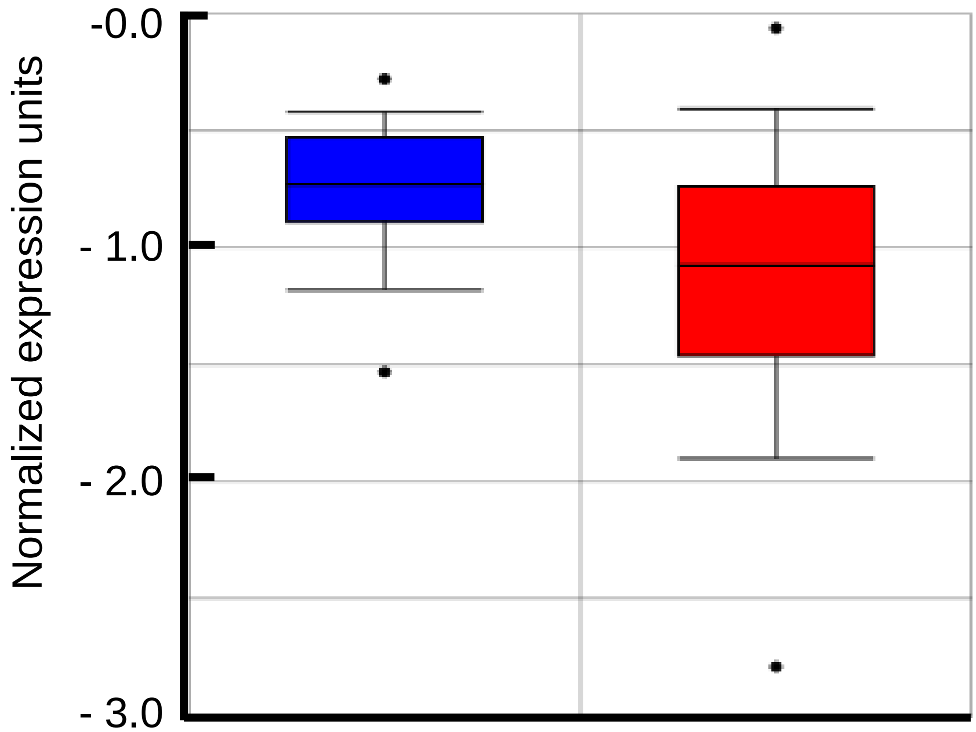

Lapointe J, Li C, Higgins JP, van de RM, Bair E *et al.* (2004) *Proc Natl Acad Sci U. S. A* 101: 811-816.

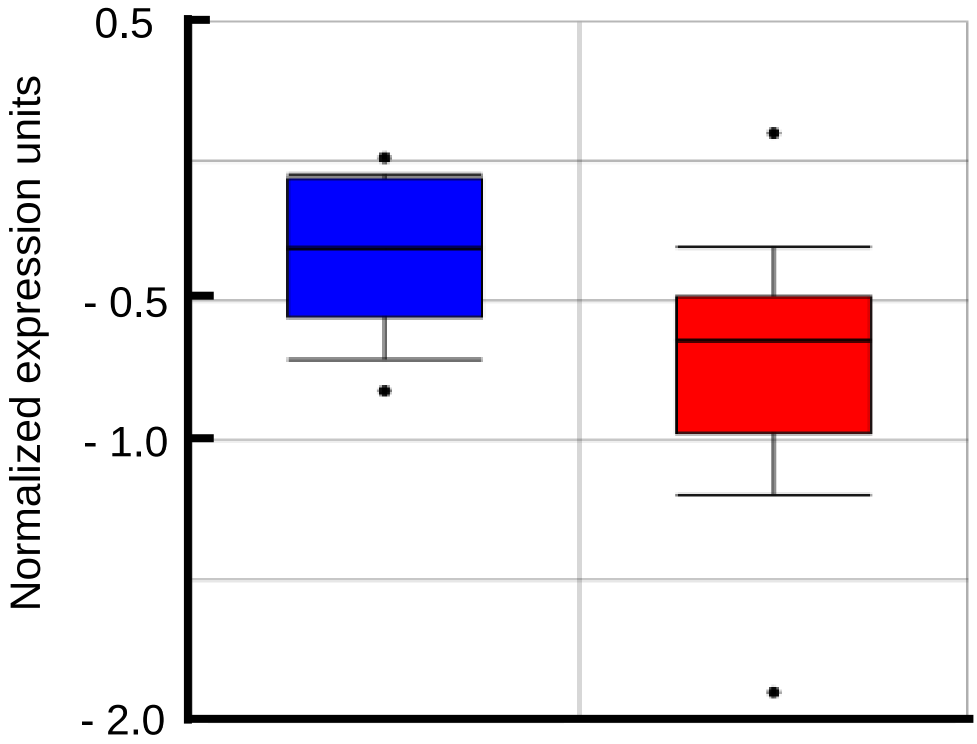

Yu YP, Landsittel D, Jing L, Nelson J, Ren B *et al.* (2004) *J Clin. Oncol.* 22: 2790-2799.

JUN

Jun oncogene

D

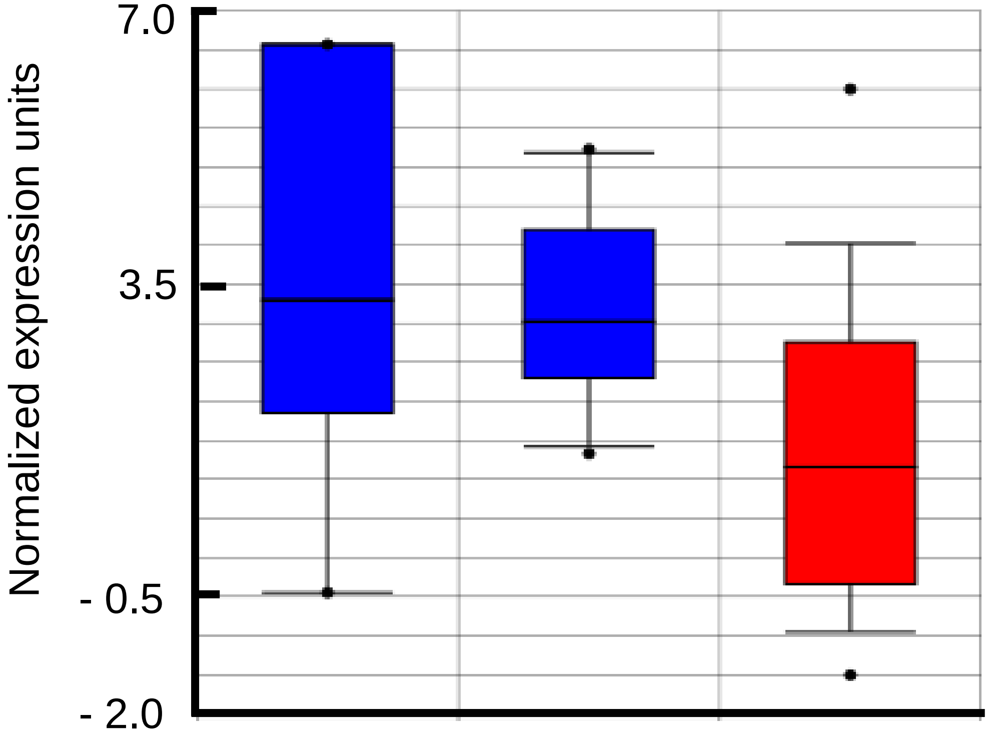

Dhanasekaran SM, Barrette TR, Ghosh D, Shah R, Varambally S *et al.* (2001) *Nature* 412: 822-826.

MYC

V-myc myelocytomatosis viral oncogene

Supplementary Fig S3

E

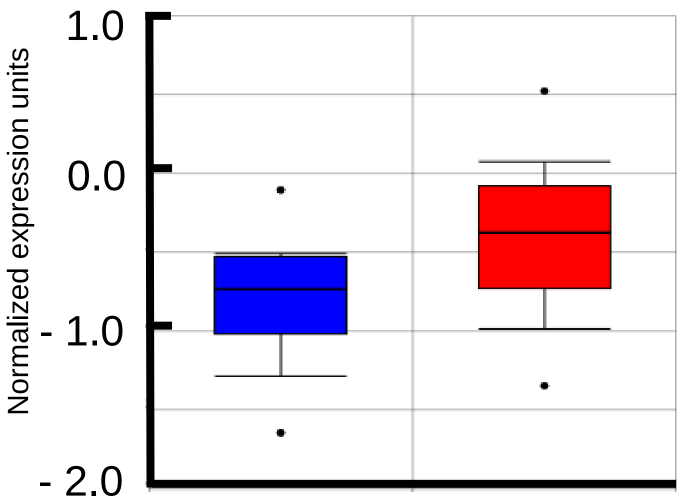

Yu YP, Landsittel D, Jing L, Nelson J, Ren B *et al.* (2004) *J Clin. Oncol.* 22: 2790-2799.

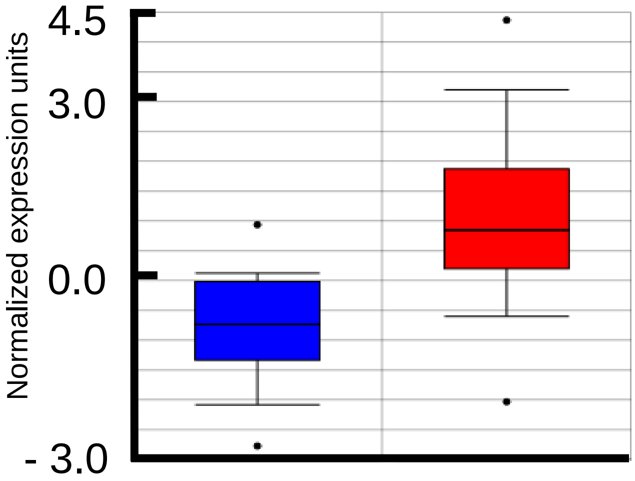

Tomlins SA, Mehra R, Rhodes DR, Cao X, Wang L *et al.* (2007) *Nat Genet* 39: 41-51.

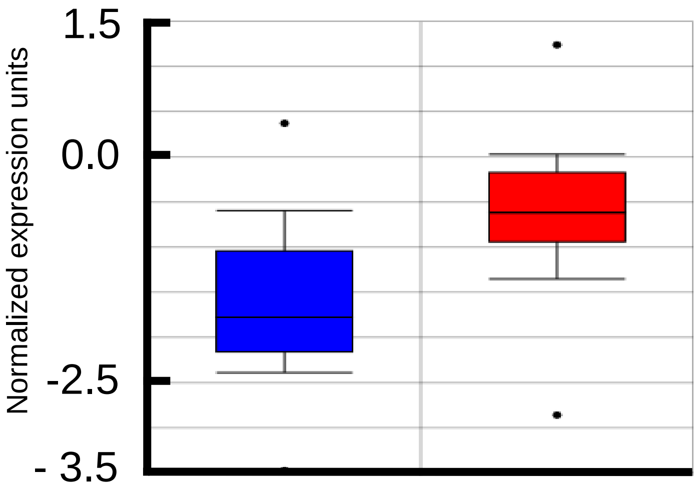

Lapointe J, Li C, Higgins JP, van de RM, Bair E *et al.* (2004) *Proc Natl Acad Sci U. S. A* 101: 811-816.

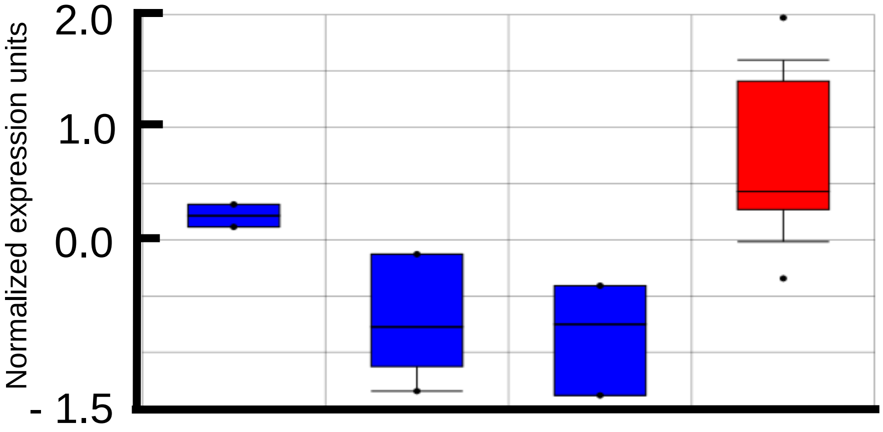

Dhanasekaran SM, Barrette TR, Ghosh D, S
hah R, Varambally S *et al.* (2001) *Nature* 412: 822-826.

Supplementary Fig S3

F

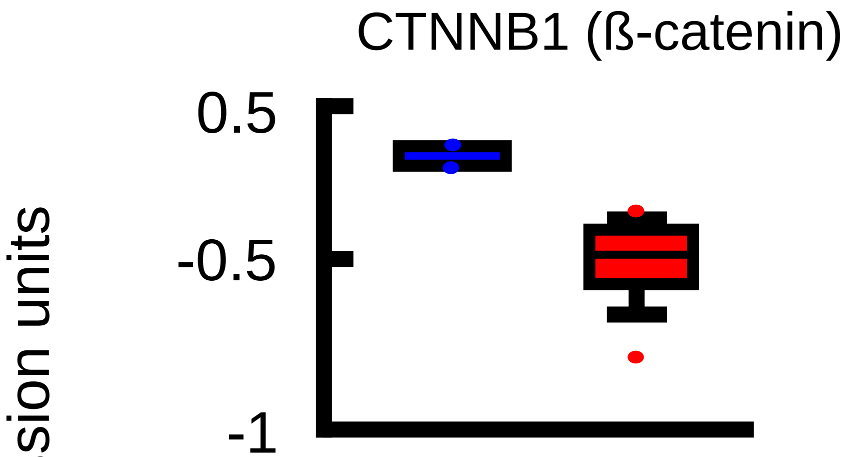

Lapointe J, Li C, Higgins JP, van de RM, Bair E *et al.* (2004) *Proc Natl Acad Sci U. S. A* 101: 811-816.

G

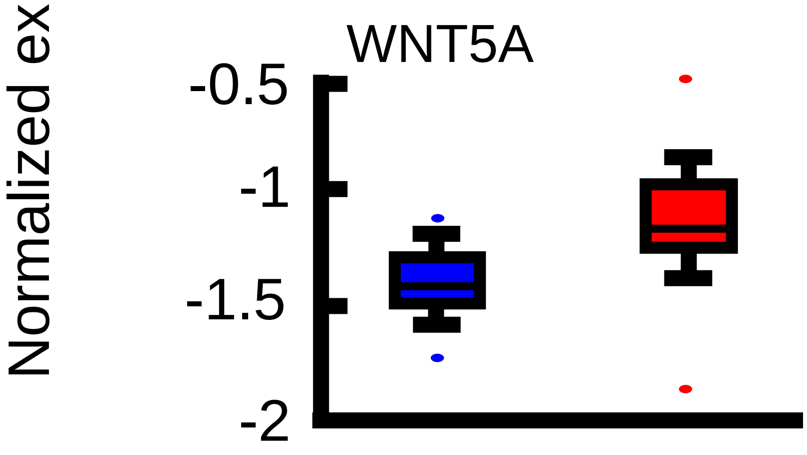

Yu YP, Landsittel D, Jing L, Nelson J, Ren B *et al.* (2004) *J Clin. Oncol.* 22: 2790-2799.
