# Supplementary material for: A Novel Role for Wnt/Ca2+ Signaling in Actin Cytoskeleton Remodeling and Cell Motility in Prostate Cancer
Source: PLoS One. 2010 May 4;5(5):e10456. doi: 10.1371/journal.pone.0010456 (PMC2864254; doi:10.1371/journal.pone.0010456)
Supplement: Methods S1 — (0.03 MB DOC) [file pone.0010456.s008.doc]

Supplementary methods: Wang, Q., Symes, A.J. et al

**Supplementary methods**

**Western blotting**

Standard Western blotting on PVDF membranes (Millipore) and indirect mmunofluorescence was performed (see supplementary methods). The following primary antibodies were used: Wnt5A (AF645; R&D System, UK), ß-catenin (ab6302; Abcam Ltd, UK) and anti-GAPDH (HyTest, Finland) and visualized using ECL-system (Pierce, UK) according to manufacturer’s instructions. Cells were lysed with RIPA buffer (50 mM Tris-HCL pH8, 150 mM NaCl, 5 mM EDTA, 1% NP40, 0.5% sodium deoxycholate, 0.1% SDS, 10% glycerol, 1 mM PMSF, 10µg/ml aprotinin, and 10µg/ml leupeptin, all from Sigma, UK). Proteins (10 µg) were separated by 10% SDS/PAGE gel electroph oresis and transferred to PVDF membranes (Millipore, UK).

**Immunocytochemistry and indirect immunofluoresence**

Cells were fixed with 4% paraformaldehyde with 0.1% Triton X-100 for 30 minutes, washed with PBS and incubated with 1% normal horse serum (Vector, UK) for 1 hour, followed by an overnight incubation with primary antibodies: Wnt5A, TIMP-3 (Ab-1) (Oncogene, UK), or MMP-14, also called MT1-MMP (MT1-MMP Ab-3, Oncogene, UK) at 4ºC. After overnight incubation with the primary antibody, coverslips were washed with PBS and probed with FITC-conjugated secondary antibody for 1 hour at RT. Cells were then washed and mounted onto glass slides with Vectashield containing DAPI (Vector, UK), and examined using a Nikon Diaphot 200 microscope (Nikon, UK) with epifluorescence filter-set for DAPI and FITC (Omega Optical, USA).

**Wound/scratch assay in prostate cell lines**

Cell lines (1542-NPTX, 1542-CP_3_TX, PC3 and DU145) were grown on glass coverslips to confluency. At the start of the assay coverslips were placed in fresh medium and scratched using a plastic pipette tip. Cells were washed with growth medium and AIP (Biomol, UK) or Wnt5A (R&D Systems, UK) added at appropriate concentrations. For AIP treated cells an unrelated synthetic peptide (NSEENDSGVTSPPAS) or sham controls were used; no difference was observed in the wound edge of sham treated or control peptide treated cells. Cells were incubated at 37^o^C and coverslips were fixed at various time points in 1ml, 4% paraformaldehyde. After overnight fixation, FITC or TRITC labelled phalloidin (Sigma, UK) was added at 0.5µg/ml and incubated with shaking at RT in dark for 3h. After this incubation the coverslips were washed 3x in phosphate buffered saline (PBS) for 5min whilst shaking and mounted on a glass slide using Vectashield (Vector) with DAPI or PI to visualize nucleic acid and stored at 4^o^C for observation using epifluorescence (using a Nikon Diaphot 200 (Nikon, UK) equipped with super high pressure mercury lamp (C-SHG1, Nikon, UK) and Omega (GlenSpectra, UK) vivid filter sets for viewing FITC or TRITC) or a confocal microscope, Leica SP2 (Leica Microsystems, Germany, with 350, 458, 476, 488, 514, 543 & 633nm laser lines). Images obtained using confocal microscopy were imported into Image J software (rsb.info.nih.gov/ij/) and processed to create *Z-stacks* presented here.

The rate of wound closure was determined by live cell imaging using Incucyte (Essen Instruments) was used for live imaging of wound closure assays to determine cell motility. PC3 cells were grown ± AIP (10µM) or Wnt5A (100ng/ml), in Image Lock 24 well plates (Essen Instruments, UK) and wound was made using the Wound Maker (Essen Instruments, UK) which makes a precise wound over a laser etched line in each well. Cells were incubated at 37^o^C and imaging was performed every hour for the subsequent 12 hours. The width of the wound (µm) is imaged over time as the laser etching on Image Lock plates provides a marker for the computer controlled camera lens to return the precise point to capture sequential images. Sequential images are composited for conversion into a movie file (Supplementary movies 1 and 2) and wound width data was saved into an Excel (Microsoft) spreadsheet for analysis. Rate of wound closure (µm/h) was plotted for the initial rate (12h) and the slope of the line was calculated by linear regression using Origin software (Microcal). PC3 cells from two different passages were used in this experiment and each condition (control or +AIP or +Wnt5A) was imaged in 6-10 individual wells. Statistical analysis for significance of difference was performed using ANOVA.

**Scanning electron microscopy and quantitation of filopodia like structures**

1542-CP_3_TX cells from two independent preparations were used for wound/scratch assay and were prepared as described for confocal microscopy above, except cells were fixed in a 4% paraformaldehyde and 2% glutaraldehyde. After fixation coverslips were post-fixed in 1% osmium tetroxide in PBS and rinsed with PBS. Following dehydration in a graded ethanol series, samples were immersed in hexamethyldisilazane, air dried prior to coating with gold and viewed using a JSM-7410 scanning electron microscope (Jeol, Japan). Coverslips were observed for the length of the wound and random images were taken for AIP treated and untreated cells at low (<2000x magnification) and high (>2000x magnification) resolution. Image files were imported into Image J software and manually analyzed for fine structures protruding from the membrane (filopodia like protrusions). For quantitation of filopodia like structures, 10-15 micrographs, comprising approximately 0.7mm of the wound length from 2 different experiments, were randomly selected. Quantification of number and length of protrusions was performed manually using Image J software from the leading edge of the wound in AIP treated and untreated cells. Multi-peak Gaussian fit of length distribution histograms was performed using Origin (Origin Lab, USA) data analysis software.
