# Supplementary material for: A Novel Role for Wnt/Ca2+ Signaling in Actin Cytoskeleton Remodeling and Cell Motility in Prostate Cancer
Source: PLoS One. 2010 May 4;5(5):e10456. doi: 10.1371/journal.pone.0010456 (PMC2864254; doi:10.1371/journal.pone.0010456)
Supplement: Table S1 — mRNA quantitation using real time PCR mRNA and comparative CT method in prostate cell lines. 2-ΔΔCT method was used to calculate relative quantities of mRNA in 1542-CP3TX relative to 1542-NPTX, using 18S rRNA as normalization controls. A. The range is determined by evaluating the expression 2-ΔΔCT with ΔΔCT + s and ΔΔCT - s, where s = SD of the ΔΔCT value. (0.03 MB DOC) [file pone.0010456.s009.doc]

**Supplementary Table S1.**

|  | Relative mRNA expression arbitrary units (range ^a^) | |
| --- | --- | --- |
| ***Gene name*** | ***1542-NPTX*** | ***1542-CP_3_TX*** |
| WNT5A | 1.00 (0.68-1.46) | 49 (42-58) |
| MMP2 | 1.00 (0.84-1.19) | 0.44 (0.37-0.53) |
| MMP14 | 1.00 (0.95-1.05) | 0.27 (0.24-0.30) |
| TIMP3 | 1.00 (0.9-1.11) | 0.01 (0.01-0.01) |
| CTNNB1 | 1.00 (0.95-1.05) | 0.39 (0.35-0.43) |
| GSK3ß | 1.00 (0.93-1.06) | 0.65 (0.58-0.72) |
| Axin | 1.00 (0.90-1.11) | 0.64 (0.57-0.72) |
| TCF4 | 1.00 (0.88-1.14) | 0.49 (0.39-0.61) |
| DVL1 | 1.00 (0.94-1.07) | 0.65 (0.58-0.72) |
