# Supplementary material for: A Novel Role for Wnt/Ca2+ Signaling in Actin Cytoskeleton Remodeling and Cell Motility in Prostate Cancer
Source: PLoS One. 2010 May 4;5(5):e10456. doi: 10.1371/journal.pone.0010456 (PMC2864254; doi:10.1371/journal.pone.0010456)
Supplement: Table S2 — Quantitation of Wnt5A expression in malignant and benign human prostate tissue using ImageJ software. DAB label, representing Wnt5A expression was quantified in an unbiased manner, by using a reproducible, semi-automated particle analysis (Analyze Particles) protocol with ImageJ software. Over 600 individual prostate tissue cores (see Materials and Methods) RGB images were converted into 16 bit grayscale (e.g., from images shown in Fig 1). The results are mean ± SE for the calculated parameters of count, total area, average size and area fraction. (0.03 MB DOC) [file pone.0010456.s010.doc]

**Supplementary Table S2**

|  |  |  |  |  |  |  |  |
| --- | --- | --- | --- | --- | --- | --- | --- |
|  |  |  |  |  |  |  |  |
|  |  |  |  |  |  |  |  |

| Prostate | Count | Total Area | Average Size | Area Fraction |
| --- | --- | --- | --- | --- |
| Malignant | 30073 ± 1027 | 1779162 ± 54348 | 64 ± 2 | 15 ± 0.5 |
| Benign | 23083 ± 935 | 998805 ± 33007 | 49 ± 1 | 8 ± 0.3 |
