# Supplementary material for: A Novel Role for Wnt/Ca2+ Signaling in Actin Cytoskeleton Remodeling and Cell Motility in Prostate Cancer
Source: PLoS One. 2010 May 4;5(5):e10456. doi: 10.1371/journal.pone.0010456 (PMC2864254; doi:10.1371/journal.pone.0010456)
Supplement: Table S3 — Genes under the transcriptional regulation of Wnt/β-catenin/TCF pathway are downregulated in the prostate cancer cell line. Downstream targets known to be under the control of canonical Wnt-signaling pathway are down-regulated in 1542-CP3TX compared to 1542-NPTX cells, possibly due to the activation of the non-canonical Wnt-signalling in these cells. (Other Wnt/β-catenin pathway targets such as PITX2, APCDD1 or JUN did not show a significant change in expression in normal and cancer cell lines.) Oligoarray data from Wang et al, Oncogene, 2007 (doi: 10.1038/sj.onc.1210472). (0.03 MB DOC) [file pone.0010456.s011.doc]

**Supplementary Table S3**

| Wnt/ß-catenin/TCF target genes | Affymetrix gene identifier no. | Fold change in 1542-CP_3_TX |
| --- | --- | --- |
| CCND1 | 208711 | -2.5 |
| CCND2 | 200951 | -5 |
| TCF7 | 205255 | -2 |
| LEF1 | 210948 | -24 |
| PPARdelta | 208044 | -2.5 |
| MMP7 | 204259 | -2 |
| CD44 | 209835 | -3 |
