# Supplementary material for: A Novel Role for Wnt/Ca2+ Signaling in Actin Cytoskeleton Remodeling and Cell Motility in Prostate Cancer
Source: PLoS One. 2010 May 4;5(5):e10456. doi: 10.1371/journal.pone.0010456 (PMC2864254; doi:10.1371/journal.pone.0010456)
Supplement: Table S4 — Applied Biosystems probe IDs for TaqMan assay. 18S rRNA (Hs99999901_s1) was used as endogenous controls. Quantitative PCR was performed using ABI Prism 7900 with MicroFluidic cards (Applied Biosystems) according to manufacturer's protocols, with each reaction containing 8ng of reverse transcribed RNA in a 2μl reaction mix. The following cycling parameters were employed: 48oC for 30 min, 95oC for 10 min, followed by 40 cycles of 95oC for 15 sec and 60oC for 15 sec. (0.03 MB DOC) [file pone.0010456.s012.doc]

**Supplementary Table S4**

| Gene | Assay ID |
| --- | --- |
| WNT5A | Hs00180103_m1 |
| TIMP3 | Hs00165949_m1 |
| MMP2 | Hs00234422_m1 |
| MMP14 | Hs00237119_m1 |
| CTNNB1 | Hs00170025_m1 |
| DVL1 | Hs00182896_m1 |
| GSK3B | Hs00275656_m1 |
| TCF4 | Hs00162613_m1 |
| AXIN1 | Hs00394718_m1 |
